# Exploring the Anti-Cancer Activity of Novel Thiosemicarbazones Generated through the Combination of Retro-Fragments: Dissection of Critical Structure-Activity Relationships

**DOI:** 10.1371/journal.pone.0110291

**Published:** 2014-10-16

**Authors:** Maciej Serda, Danuta S. Kalinowski, Nathalie Rasko, Eliška Potůčková, Anna Mrozek-Wilczkiewicz, Robert Musiol, Jan G. Małecki, Mieczysław Sajewicz, Alicja Ratuszna, Angelika Muchowicz, Jakub Gołąb, Tomáš Šimůnek, Des R. Richardson, Jaroslaw Polanski

**Affiliations:** 1 Institute of Chemistry, University of Silesia, Katowice, Silesia, Poland; 2 Department of Pathology and Bosch Institute, University of Sydney, Sydney, New South Wales, Australia; 3 Department of Biochemical Sciences, Charles University in Prague, Faculty of Pharmacy in Hradec Králové, Hradec Králové, Czech Republic; 4 A. Chełkowski Institute of Physics and Silesian Interdisciplinary Centre for Education and Research, University of Silesia, Katowice, Silesia, Poland; 5 Department of Immunology, Medical University of Warsaw, Warsaw, Mazovia, Poland; 6 Institute of Physical Chemistry, Polish Academy of Sciences, Warsaw, Mazovia, Poland; University of Melbourne, Australia

## Abstract

Thiosemicarbazones (TSCs) are an interesting class of ligands that show a diverse range of biological activity, including anti-fungal, anti-viral and anti-cancer effects. Our previous studies have demonstrated the potent *in vivo* anti-tumor activity of novel TSCs and their ability to overcome resistance to clinically used chemotherapeutics. In the current study, 35 novel TSCs of 6 different classes were designed using a combination of retro-fragments that appear in other TSCs. Additionally, di-substitution at the terminal N4 atom, which was previously identified to be critical for potent anti-cancer activity, was preserved through the incorporation of an N4-based piperazine or morpholine ring. The anti-proliferative activity of the novel TSCs were examined in a variety of cancer and normal cell-types. In particular, compounds **1d** and **3c** demonstrated the greatest promise as anti-cancer agents with potent and selective anti-proliferative activity. Structure-activity relationship studies revealed that the chelators that utilized “soft” donor atoms, such as nitrogen and sulfur, resulted in potent anti-cancer activity. Indeed, the *N*,*N*,*S* donor atom set was crucial for the formation of redox active iron complexes that were able to mediate the oxidation of ascorbate. This further highlights the important role of reactive oxygen species generation in mediating potent anti-cancer activity. Significantly, this study identified the potent and selective anti-cancer activity of **1d** and **3c** that warrants further examination.

## Introduction

Iron is an essential element that is necessary for a number of cellular processes, such as cellular proliferation [1], [2], [3]. In fact, the iron-containing enzyme, ribonucleotide redutase, is involved in the rate-limiting step of DNA synthesis and is responsible for the conversion of ribonucleotides to their deoxyribonucleotide counterparts [4], [5]. Importantly, alterations in the iron metabolism of cancer cells relative to their normal counterparts have highlighted the potential of iron chelation therapy to act as a novel treatment avenue. Cancer cells demonstrate a higher requirement for iron than normal cells and this is emphasized by the increased expression of the transferrin receptor 1 (TfR1), that takes up iron from the iron transport protein, transferrin (Tf), on the cell surface [6], [7], [8]. Additionally, the expression of iron-dependent enzyme, ribonucleotide reductase, is markedly higher in tumor cells than normal cells [9]. These factors render tumor cells more sensitive to iron chelation.

Although iron chelators (*e.g.*, desferrioxamine; DFO) have been clinically utilized for the treatment of iron overload disease [1], [3], novel thiosemicarbazone (TSC) chelators have been widely investigated as potential anti-cancer agents [10], [11], [12], [13], [14], [15], [16], [17], [18], [19], [20], [21]. Although the molecular mechanisms involved in the activity of TSCs have not been completely elucidated, a number of modes of action have been reported [3], [15], [21], [22], [23]. This includes: *(* ***1*** *)* the inhibition of cellular iron uptake from Tf [10], [13], [18]; *(* ***2*** *)* the mobilization of iron from cells [10], [13], [18]; *(* ***3*** *)* the inhibition of the ribonucleotide reductase activity [24], [25]; *(* ***4*** *)* the up-regulation of the metastasis suppressor protein, N-myc downstream regulated gene 1 [26], [27], [28], [29]; and *(* ***5*** *)* the formation redox active metal complexes that produce reactive oxygen species (ROS) [10], [15], [21], [23], [30]. This latter mechanism is significant, especially as studies have demonstrated the important role of ROS generation in increasing the selective activity of chelators against tumor cells [10], [15], [21].

**Figure 1:**
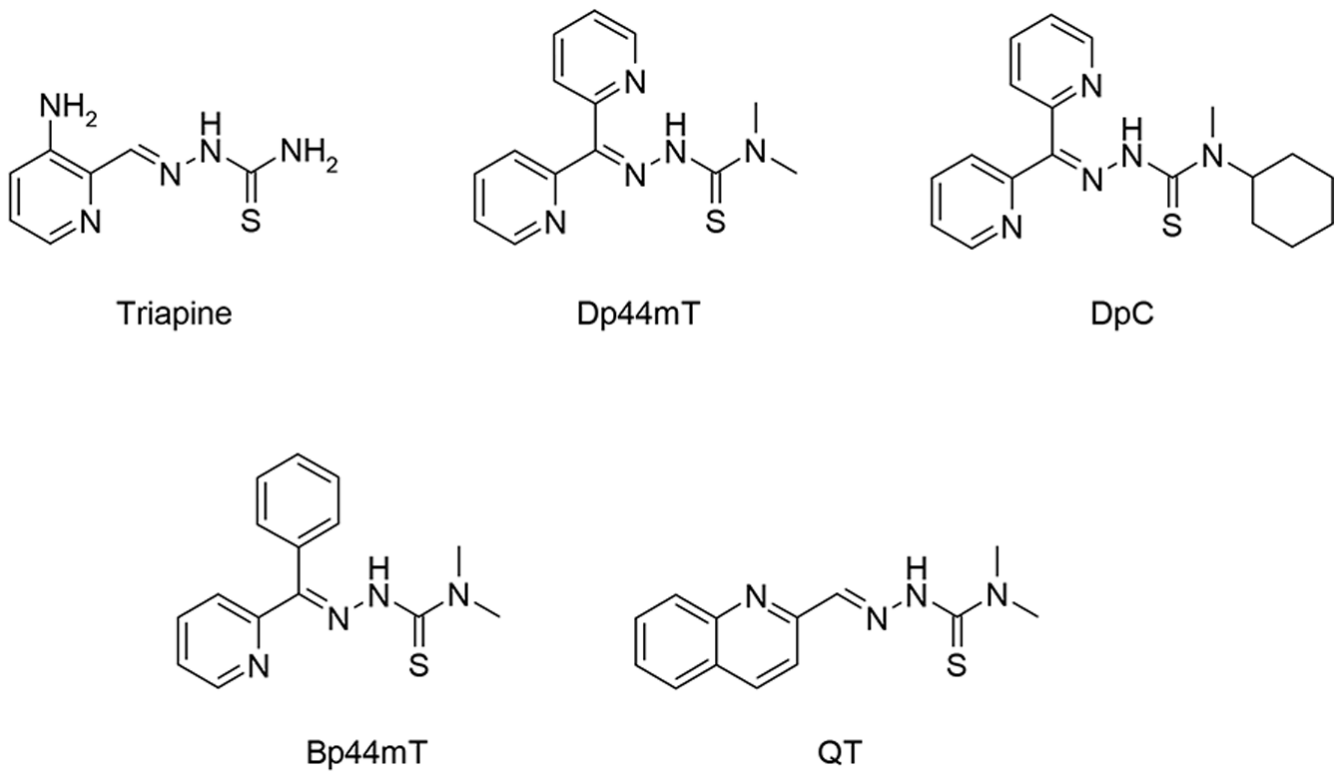
Chemical structures of the chelators, Triapine, di-2-pyridylketone 4,4-dimethyl-3-thiosemicarbazone (Dp44mT), di-2-pyridylketone 4-cyclohexyl-4-methyl-3-thiosemicarbazone (DpC), 2-benzoylpyridine 4,4-dimethyl-3-thiosemicarbazone (Bp44mT) and quinoline thiosemicarbazone (QT).

The TSC, 3-aminopyridine-2-carboxaldehyde thiosemicarbazone (Triapine; Fig. 1), has been examined in >20 Phase I and II clinical trials as a novel cancer chemotherapeutic [11], [31], [32], [33], [34], [35], [36], [37], [38], [39], [40], [41]. Although clinical trials using Triapine have generally demonstrated limited anti-tumor activity [36], [37], [38], [40], other studies have shown positive results in locally advanced cervical and vaginal cancers when co-administered with cisplatin and radiochemotherapy [33], [34]. Notable side effects observed upon Triapine administration include methemoglobin formation and hypoxia [35], [39], [41] and these problems have necessitated the development of other more active and selective TSCs with potent anti-cancer activity.

Several classes of TSCs have been developed as potential anti-proliferative agents (Fig. 1), a number of which show marked and selective anti-tumor activity both *in vitro* [10], [15], [21], [42] and *in vivo* [12], [21], [26], [42]. For example, a five day treatment with di-2-pyridylketone 4,4-dimethyl-3-thiosemicarbazone (Dp44mT; Fig. 1) at 0.4 mg/kg in mice reduced the growth of a murine M109 lung carcinoma to 47% of the control [21]. Additionally, Dp44mT showed potent and selective anti-tumor activity *in vitro* and *in vivo* against a range of human tumor xenografts [42] and was able to form redox active metal complexes that generate ROS [15], [21], [30]. Although this TSC showed great potential, it demonstrated cardiac fibrosis at high, non-optimal doses [42]. Thus, further investigations into Dp44mT analogs were necessary and have resulted in the development of di-2-pyridylketone 4-cyclohexyl-4-methyl-3-thiosemicarbazone (DpC; Fig. 1) [12], [26]. DpC has demonstrated selective *in vitro* and *in vivo* anti-tumor activity by both the intravenous [12], [26] and oral routes [12] and is currently being further evaluated for entrance into clinical trials. Recently, other TSCs have also been shown to have a novel application as photodynamic therapy enhancers [43].

We have previously examined a variety of quinolone-based TSCs that demonstrate *in vitro* anti-proliferative activity [16]. In the current investigation, we synthesized 35 novel TSCs of 6 classes that were designed by the combination of active fragments present in previously reported analogs [16], [44]. Earlier studies indicated that di-substitution at the terminal (N4) nitrogen is crucial for effective anti-cancer activity [12], [21]. In the present study, we have preserved di-substitution at the N4 atom through the construction of an N4-based piperazine or morpholine ring, a fragment that is present in several active TSCs [45], [46]. The anti-proliferative activity and selectivity of these novel TSCs was examined *in vitro* against human cancer cell-types and normal human dermal fibroblast (NHDF) cells. Those series that demonstrated the ability to form redox active iron complexes and mediate the oxidation of ascorbate showed potent anti-proliferative activity, highlighting the importance of ROS generation in their anti-cancer activity.

## Materials and Methods

The reagents were purchased from Sigma-Aldrich (St. Louis, MO, USA), ACROS Organics (Geel, Belgium) or Princeton Chemicals Ltd (Luton, Bedfordshire, UK). Silica gel 60 (0.040-0.063 mm; Merck, Darmstadt, Germany) was used for column chromatography. Thin layer chromatography (TLC) experiments were performed on alumina-backed silica gel 40 F_254_ plates (Merck). The plates were illuminated under UV (254 nm) and evaluated in iodine vapor. The melting points were determined on an Optimelt MPA100 instrument (Stanford Research Systems, Sunnyvale, CA, USA) and are uncorrected. High resolution-mass spectrometry (HRMS) analysis was performed for all new compounds on a Finnigan MAT95 spectrometer (Thermo Fisher Scientific, Bremen, Germany) or on a Mariner ESI-TOF spectrometer (Applied Biosystems, Thermo Fisher Scientific).

All ^1^H- and ^13^C-NMR spectra were recorded on a Bruker AM-400 spectrometer (399.95 MHz for ^1^H; 99.99 MHz for ^13^C; BrukerBioSpin Corp., Coventry, UK). Chemical shifts are reported in ppm against the internal standard, Si(CH_3_)_4_. Easily exchangeable signals were omitted when diffuse. Syntheses were performed on a CEM-DISCOVERY microwave reactor (CEM Corporation, Matthews, NC, USA) with temperature and pressure control.

Log *P*_calc_ values were calculated using ChemDraw 12 (Perkin-Elmer, Waltham, MA, USA) by performing Crippen's fragmentation [47], Viswanadhan's fragmentation [48] and Broto's method [49] and then calculating the average log *P*_calc_.

### Synthesis of Thiosemicarbazide Precursors (a–f)

#### General method I

**Figure 2:**
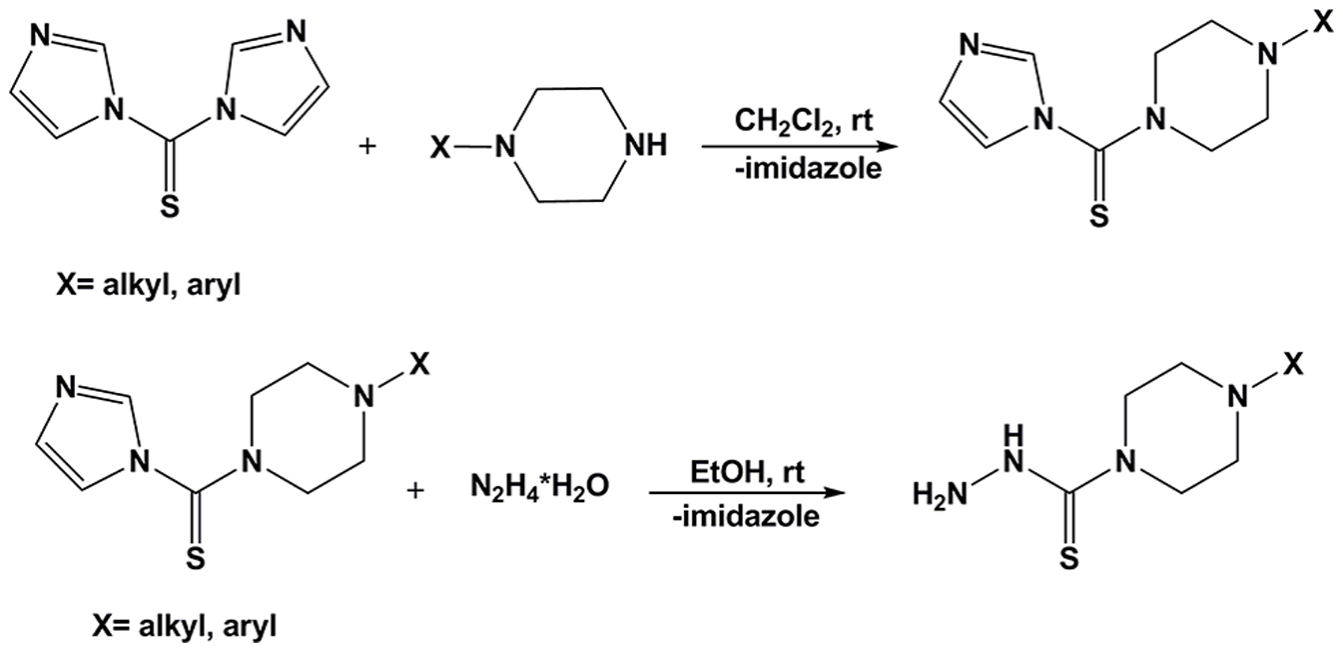
Synthetic route to yield thiosemicarbazides, a–e.

*N*-alkyl or *N*-aryl piperazine (6 mmol) was added to a solution of 1,1′-thiocarbonyldiimidazole (6 mmol; 1.068 g) in 25 mL of dichloromethane and the reaction mixture was stirred for 24 h at room temperature. The organic solvent was separated and extracted 3 times using water, then dried over anhydrous magnesium sulfate, filtered and concentrated to provide the crude piperazine derivatives. These crude products were used in the next step without further purification. These intermediates were added to a solution of hydrazine hydrate in 25 mL of ethanol at room temperature. The reaction mixture was refluxed for 3 h and cooled to obtain a white precipitate which was collected *via* filtration as the final product (Fig. 2). All obtained thiosemicarbazides were crystallized from methanol. See File S1 for structural characterization details and the crystal structure (Fig. S1 in File S1) and data (Table S1 in File S1) of 4-ethylpiperazine-1-carbothiohydrazide (**a**).

#### General method II

**Figure 3:**
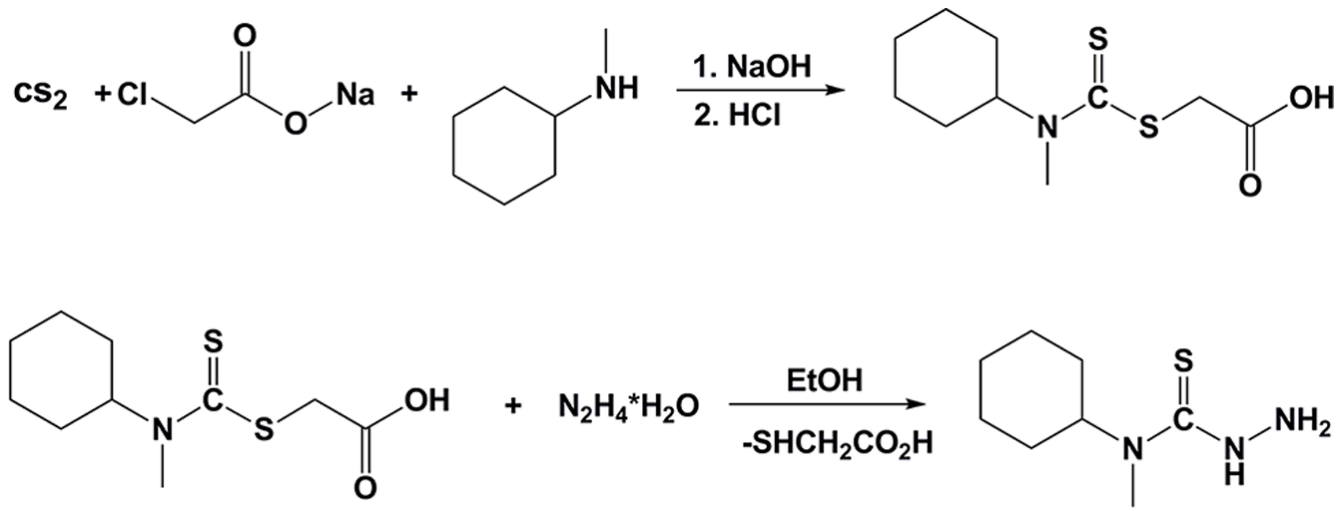
Synthetic route to yield the thiosemicarbazide, f.

Carbon disulfide (CS_2_) (0.2 mol, 12.06 mL) was added dropwise over 15 min to *N*-methylcyclohexylamine (0.2 mol, 26.3 mL) in NaOH solution (0.8 M, 250 mL). The reaction mixture was intensively stirred for 20 min and then sodium chloroacetate (0.2 mol) was added to the solution and allowed to stir for 15 h. The reaction mixture was neutralized with concentrated HCl (20 mL) to give the carboxymethylthiocarbamate intermediate as a white precipitate. The obtained carboxymethylthiocarbamate intermediate (0.08 mol) was then reacted with hydrazine hydrate (0.08 mol) in water (10 mL). This reaction mixture was gently refluxed for 2 h to give white crystals of the thiosemicarbazide (Fig. 3). The final product was crystalized from methanol-water (1/1, *v/v*).

### Preparation of TSC Derivatives

**Figure 4:**
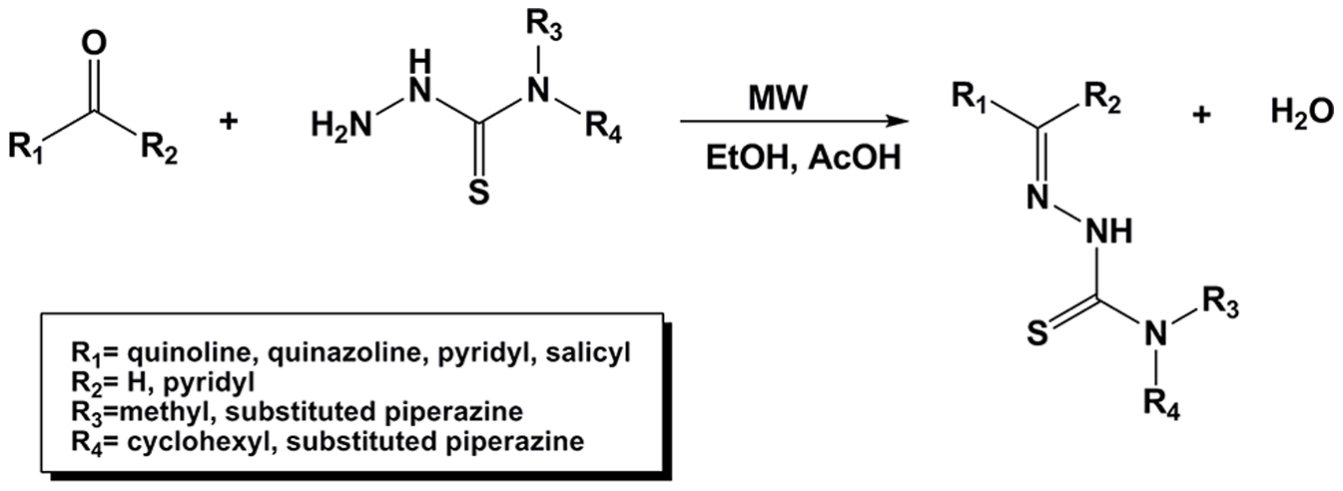
Synthetic route to yield the final thiosemicarbazones.

The heteroaromatic TSC analogs were synthesized by reacting the respective heteroaromatic ketone or carbaldehyde and thiosemicarbazide under microwave irradiation. Equimolar quantities of the appropriate thiosemicarbazide (0.5 mmol) and carbonyl compound (0.5 mmol) were dissolved in 4 mL of EtOH with the addition of 0.1 mL of acetic acid as catalyst (Fig. 4). The resulting mixture was heated in a microwave reactor at 83°C/30 min (max. microwave power 50 W). After cooling, the precipitated solid was filtered and washed with ether. The final product was purified using crystallization (from ethanol or methanol) or column chromatography. See File S1 for structural characterization details, the crystal structure (Fig. S2 in File S1) and data (Table S1 in File S1) of *Z*-*N*′-(di(pyridin-2-yl)methylene)-4-(pyridin-2-yl)piperazine-1-carbothiohydrazide (**1d**) and isosbestic curves (Fig. S3 in File S1).

### HPLC Purity Data

The final purity of the TSCs was determined using the following general conditions: Gynkotek HPLC System (Gynkotek); Pump T580; Autosampler GINA50 (Gynkotek); Detector DAAD UVD340U; Column: Hilic Kinetex 100 Å (Phenomenex, Torrance, CA, USA); Flow: 0.5 mL/min (0–1 min), 0.5–1.2 mL/min (1–3 min), 1.2 mL/min (3–7 min), 1.2–0.5 mL/min (7–8 min), 90% CH_2_Cl_2_, 10% CH_3_OH; UV at 250 nm; Software: Chromeleon (Thermo Scientific, Waltham, MA, USA). See File S1 for HPLC purity data (Table S2 in File S1).

### Cell Culture

The human cancer cell types (neuroepithelioma (SK-N-MC), colon cancer (HCT116 with wild-type p53 (p53^+/+^)), Burkitt's lymphoma (Raji) and cervical carcinoma (HeLa)) cells and normal human dermal fibroblasts (NHDF) were obtained from the American Type Culture Collection (ATCC, Manassas, VA, USA). HCT116 null p53 (p53^−/−^) cells were obtained from the Maria Sklodowska-Curie Memorial Cancer Center and Institute of Oncology, Poland.

The SK-N-MC cells were cultured in minimum essential medium (MEM; Life Technologies, Grand Island, New York, USA) containing 10% (v/v) fetal bovine serum (FBS; Life Technologies), 1 mM sodium pyruvate (Life Technologies), 1% (v/v) non-essential amino acids (Life Technologies), 2 mM L-glutamine (Life Technologies), 100 U/mL penicillin (Life Technologies), 100 µg/mL streptomycin (Life Technologies) and 0.28 µg/mL fungizone (Bristol Myers Squibb Pharmaceuticals, Montreal, Canada). The HCT116 and NHDF cells were grown in Dulbecco's modified Eagle's medium (DMEM; Sigma-Aldrich) supplemented with 12% (v/v) heat-inactivated FBS (HCT116; Life Technologies) or 15% (v/v) FBS (NHDF; Life Technologies), 100 µg/mL of gentamicin (Polfa Warszawa S.A., Warsaw, Poland), 100 µg/mL of streptomycin (Polfa) and 100 U/mL of penicillin (Polfa). The Raji and HeLa cells were cultured in Roswell Park Memorial Institute medium (RPMI-1640; Sigma-Aldrich) with the addition of 10% (v/v) heat-inactivated FBS (Life Technologies) and the supplements described for SK-N-MC cells above. All cell lines were cultured under standard conditions at 37°C in a humidified atmosphere at 5% CO_2_ and were subcultured every 3–4 days as required.

### Proliferation Assay

The cells were seeded in 96-well plates (SK-N-MC: 1.5×10^4^ cells/well; HeLa: 5.0×10^3^ cells/well; Raji: 3.0×10^3^ cells/well; HCT116: 3.5×10^3^ cells/well; NHDF: 3.0×10^3^ cells/well) 24 h prior to the addition of the novel compounds. The assays were performed using a 72 h (SK-N-MC, Raji, HeLa) or 96 h (HCT 116, NHDF) incubation period with the agents at 37°C. These seeding and growth conditions were utilized so that the cells did not come to confluence during the incubation periods. Additionally, DFO and Dp44mT were included as positive controls in all experiments as their activity is well characterized [21], [42], [50].

Chelator stock solutions were prepared in DMSO and diluted in media so that the final [DMSO] <0.05%. The results were expressed as a percentage of the control and the resulting IC_50_ values were calculated using GraphPad Prism 5 (GraphPad Software, Inc., La Jolla, CA, USA). The IC_50_ was defined as the concentration necessary to reduce the absorbance to 50% of the untreated control. Each individual compound was tested in triplicate in a single experiment, with each experiment being repeated three times. After incubation of HCT 116 and NHDF cells with the tested compounds, 20 µL of the CellTiter 96 Aqueous One Solution - MTS (Promega, Madison, WI, USA) solution was added to each well (with 100 µL of DMEM without phenol red) and incubated for 1 h/37°C. The optical density of the samples was analyzed at 490 nm.

MTT (3-[4,5-dimethylthiazol-2-yl]-2,5-diphenyltetrazolium bromide) was used to evaluate the anti-proliferative effects of the chelators in SK-N-MC and Raji cells. Following incubation with the investigated compounds, 10 µL of MTT (5 mg/mL in PBS; Sigma-Aldrich) was added to each well. After a 2 h (SK-N-MC) or 4 h (Raji) incubation, the plates were centrifuged, and the cells were lysed with 100 µL of 10% SDS-50% isobutanol in 0.01 M HCl (SK-N-MC) or DMSO (Raji). The absorbance was measured at 570 nm. The anti-proliferative effects of these novel agents on HeLa cells were estimated using crystal violet staining (0.5% crystal violet solution for 10 min). Finally, the wells were rinsed with water and the cells were lysed with 2% SDS. The optical density of the samples was analyzed at 595 nm.

### Labeling of Transferrin with ^59^Fe

The iron transport protein, Tf (Sigma-Aldrich), was labeled with ^59^Fe (PerkinElmer Life and Analytical Sciences, Boston, MA) to form ^59^Fe_2_-Tf using standard methods [6], [7]. Unbound ^59^Fe was removed by passage through a Sephadex G25 column and was followed by exhaustive dialysis [6], [7].

### Effect of the Chelators on Mobilizing Cellular ^59^Fe

To examine the ability of the novel chelators to mobilize ^59^Fe from SK-N-MC cells, ^59^Fe efflux experiments were performed using established techniques [50], [51]. The monolayer of SK-N-MC cells was prelabeled for 3 h at 37°C in MEM containing ^59^Fe_2_-Tf (0.75 µM). Cells were then washed four times with ice-cold PBS and incubated for a further 3 h at 37°C with medium alone (the control) or medium containing the chelator (25 µM). After this incubation, the overlying medium that contained the released ^59^Fe was separated from the cells using a Pasteur pipette. Radioactivity was measured in both the cells and supernatant using a γ-scintillation counter (Wallac Wizard 3, Turku, Finland). In these studies, the novel ligands were compared to the previously characterized chelators, DFO and Dp44mT, as their ability to mobilize cellular ^59^Fe has been extensively examined in these cells [10], [12], [17], [18], [50].

### Effect of Chelators at Preventing Cellular ^59^Fe Uptake

The ability of the chelators to prevent the cellular uptake of ^59^Fe from ^59^Fe_2_-Tf was examined using standard methods [10], [14], [17], [18], [50]. A monolayer of SK-N-MC cells was incubated with medium containing ^59^Fe_2_-Tf (0.75 µM) and the chelator (25 µM) for 3 h at 37°C. The medium was then removed and the cells were washed four times with ice-cold PBS. The cells were then incubated for 30 min at 4°C with the general protease, Pronase (1 mg/mL; Sigma-Aldrich), to remove membrane-bound ^59^Fe. The cells were removed using a plastic spatula and centrifuged at 14,000 rpm/1 min to separate internalized from membrane-bound ^59^Fe. The cell pellet was resuspended in 1 mL of PBS and the internalized ^59^Fe was measured on a γ-scintillation counter. Internalized ^59^Fe uptake was calculated as a percentage of the control (medium alone). Again, the novel ligands were compared to DFO and Dp44mT as their ability to inhibit cellular ^59^Fe uptake has been extensively characterized using SK-N-MC cells [10], [14], [17], [18], [50].

### Ascorbate Oxidation Assay

The ability of the iron complexes of the novel ligands to mediate the oxidation of the physiological substrate, ascorbate, was examined using established methods [10], [18], [22], [52], [53]. Ascorbic acid (100 µM) was prepared immediately prior to each experiment and was incubated in the presence of Fe^III^ (10 µM; as FeCl_3_), the chelator (1–60 µM) and a 50-fold molar excess of citrate (500 µM). The absorbance at 265 nm was measured after 10 and 40 min and the difference in absorbance at these time points was calculated. The results of these experiments were expressed as iron-binding equivalents (IBE) due to the different denticity of the chelators examined. The chelators, Dp44mT, DFO and ethylenediaminetetraacetic acid (EDTA) were used as controls as the ability of their iron complexes to oxidize ascorbate has been extensively characterized [10], [15], [52], [53].

### Statistical Analysis

Data was expressed as mean ±S.D. of at least 3 experiments. Statistical analyses were performed using Prism v6 (GraphPad Software, Inc.) implementing a one-way ANOVA with Bonferroni's post-hoc test.

## Results and Discussion

### Drug Design

Molecular properties such as molecular weight (MW) and calculated octanol-water partition coefficient (log *P*_calc_) are key factors in the successful development of drug candidates [54], [55]. In general, the average MW and log *P*_calc_ of drugs that are launched on the market are 300–450 Da and 2–4, respectively [56], [57]. Considering this, the clinically trialed TSC, Triapine (MW: 195 Da; log *P*_calc_: 0.761), represents an interesting lead molecule with a considerable reserve in MW and log *P*_calc_ for modification.

Identifying functional fragments for drug design is a complex problem that involves different approaches, including those with an experimental and theoretical basis. The latter consist of a variety of methods among which are those identifying advantageous sub-structures, scaffolds and/or linkers on the basis of previously reported compounds. Alternatively, the fragmentation of organic molecules into smaller moieties is an important method in retrosynthetic analysis and has inspired various pseudo-retrosynthetic approaches [58]. This has identified fragments that may be useful for drug design. For example, the di-2-pyridyl [12], [15], [21], [59], quinolinyl [60], piperazinyl [60], [61], [62], morpholinyl [63] and quinoxalinyl [64] motifs are common fragments in other anti-cancer agents, which have been incorporated into the design of the novel TSCs reported herein.

**Figure 5:**
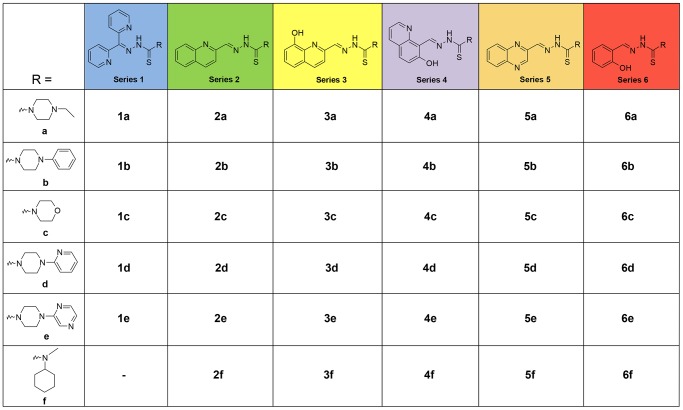
Chemical structures of the thiosemicarbazone chelators of series 1–6.

In the current study, the main rationale underlying our approach was that the newly synthesized TSCs should preserve the potent anti-cancer activity of their TSC precursors, while retaining appropriate MW and log *P*_calc_ values to show substantial promise as drug candidates for pharmaceutical development. We used a combination of retro-fragments that appear in other TSC precursors [15], [16], [21], [44], [59] and other anti-cancer agents [60], [61], [62], [63], [64]. Additionally, di-substitution at the terminal N4 atom was preserved through the construction of an N4-based piperazine or morpholine ring (Fig. 5), a moiety that is observed in several potent TSCs [45], [46].

### Synthesis of Novel Ligands

The thiosemicarbazide precursors, **a–f** (Fig. 5), were synthesized from commercially available reagents in a two-step process that gave moderate to high yields (47–95%). The treatment of bis(imidazole)thioketone with the appropriate piperazine, followed by the reaction with hydrazine hydrate gave the *N*-substituted piperazine-based thiosemicarbazides in high yield (File S1). The final thiosemicarbazone series, **1–6** (Fig. 5), were synthesized in moderate to high yield (58–96%) by Schiff-base condensation of the appropriate ketone/aldehyde with the prepared thiosemicarbazides. The purity of the thiosemicarbazones was confirmed by HPLC and was >95% (see Table S2 in File S1).

### Anti-Proliferative Activity of the Novel Thiosemicarbazones Against a Variety of Cancer Cell-Types

The ability of the novel thiosemicarbazone series **1–6** to inhibit the cellular proliferation of tumor cells was examined in a variety of cancer cell-types (Table 1), including human p53 wild-type and null colon cancer (HCT116 p53^+/+^ and HCT116 p53^−/−^, respectively), Burkitt's lymphoma (Raji), human cervical carcinoma (HeLa) and neuroepithelioma (SK-N-MC) cancer cells. The ability of these novel agents to selectivity target cancer cells was assessed by examining their effects on the cellular proliferation of a mortal cell-type, namely normal human dermal fibroblast (NHDF) cells. The anti-proliferative activity of series **1–6** is presented in Table 1 and is also represented as color maps (see Fig. S4 in File S1). The effects of these novel ligands were then compared to the following chelators that were used as positive controls: *(* ***1*** *)* DFO, that is clinically used for the treatment of iron overload disease [1], [3] and *(* ***2*** *)* Dp44mT, a chelator with potent anti-proliferative activity *in vitro* and *in vivo* [15], [21].

**Table 1:** Anti-proliferative activity (IC_50_ values) of the novel thiosemicarbazones in comparison to DFO and Dp44mT in several tumor cell-types and normal human dermal fibroblast (NHDF) cells.

	IC_50_ (µM)					
Chelator	HCT116 p53^+/+^	HCT116 p53^−/−^	Raji	HeLa	SK-N-MC	NHDF
DFO	>25	>25	4.74±1.80	14.34±0.96	15.06±5.2	>25
Dp44mT	0.002±0.001	0.005±0.002	0.007±0.001	0.04±0.01	0.012±0.001	15.38±5.06
**1a**	0.020±0.002	0.040±0.003	0.08±0.02	0.48±0.31	0.18±0.09	0.017±0.004
**1b**	0.0008±0.0001	0.0008±0.0001	0.0003±0.00003	0.57±0.16	0.05±0.01	0.0017±0.0007
**1c**	0.014±0.005	0.014±0.006	0.003±0.004	2.21±0.47	1.21±0.35	0.016±0.006
**1d**	0.002±0.008	0.009±0.001	0.0013±0.0001	0.03±0.02	0.02±0.01	0.16±0.04
**1e**	0.038±0.003	0.031±0.003	0.04±0.02	0.72±0.07	0.07±0.05	0.014±0.003
**2a**	1.44±0.69	3.55±1.23	0.06±0.02	0.43±0.16	2.54±0.86	9.62±1.80
**2b**	0.042±0.003	0.09±0.04	0.04±0.01	0.20±0.07	0.33±0.02	0.033±0.007
**2c**	1.49±0.02	1.56±1.12	0.06±0.01	1.75±0.33	1.40±0.69	3.27±0.88
**2d**	0.39±0.15	0.27±0.16	0.05±0.02	5.14±2.87	0.52±0.16	0.032±0.008
**2e**	0.088±0.044	0.48±0.15	0.29±0.11	1.56±0.44	0.48±0.10	9.44±3.84
**2f**	0.026±0.001	0.035±0.003	0.81±0.10	0.94±0.45	0.82±0.19	0.015±0.005
**3a**	9.25±1.42	8.83±0.63	0.20±0.02	3.41±1.46	0.82±0.61	15.8±4.6
**3b**	4.18±1.46	4.67±2.04	0.19±0.02	8.45±2.09	>6.25	8.88±1.07
**3c**	0.004±0.002	0.017±0.006	0.02±0.01	0.18±0.07	>6.25	10.6±3.5
**3d**	8.41±1.78	8.46±0.25	0.12±0.04	6.59±0.13	>6.25	10.2±2.4
**3e**	0.05±0.03	5.01±2.55	0.04±0.02	2.39±0.02	>6.25	12.1±3.6
**3f**	3.11±1.92	4.35±1.35	0.01±0.04	2.57±0.46	>6.25	5.36±1.99
**4a**	20.2±0.3	20.3±0.9	3.35±1.11	11.3±1.6	5.09±0.47	12.1±4.2
**4b**	7.45±0.66	8.88±2.53	2.23±1.20	4.38±0.95	4.46±0.78	10.1±1.2
**4c**	20.1±0.3	19.8±4.9	3.53±1.87	10.2±1.2	4.97±0.95	>25
**4d**	9.15±1.19	6.84±5.48	1.55±0.40	2.73±0.55	4.54±1.60	10.1±2.7
**4e**	10.1±0.7	9.44±1.35	4.55±2.52	3.49±0.04	4.17±1.44	9.56±1.44
**4f**	19.0±0.5	18.9±3.6	0.46±0.23	13.3±1.0	>6.25	9.18±3.68
**5a**	6.15±1.28	8.72±1.64	0.11±0.02	1.12±0.03	ND	2.73±1.77
**5b**	3.92±0.74	2.24±1.47	0.50±0.05	6.05±2.73	3.44±1.12	9.61±0.90
**5c**	2.03±1.24	5.84±1.65	0.24±0.04	0.86±0.09	2.87±1.25	11.6±1.8
**5d**	9.4±0.8	9.41±1.04	0.49±0.09	1.78±0.77	2.33±0.09	7.13±0.82
**5e**	5.73±0.75	8.49±3.05	0.27±0.01	3.77±1.71	1.98±0.68	15.8±5.1
**5f**	1.24±0.45	1.02±0.54	0.72±0.29	4.09±2.07	2.04±0.34	0.50±0.21
**6a**	>25	>25	10.2±6.6	22.1±5.4	>6.25	>25
**6b**	6.9±2.3	8.86±1.46	1.96±0.85	5.80±0.48	5.13±0.51	13.5±4.8
**6c**	15.4±1.7	16.4±3.1	3.79±0.08	18.2±1.3	>6.25	>25
**6d**	9.7±2.5	10.6±1.2	2.60±0.33	16.4±4.5	5.42±0.64	18.4±1.9
**6e**	10.9±0.7	10.7±1.0	9.83±2.05	19.2±2.5	>6.25	>25
**6f**	>25	>25	0.69±0.51	10.1±3.6	>6.25	>25

Results are mean ±SD (3 experiments).

As expected from our previous studies [10], [14], [17], [18], [50], the control chelator, DFO, demonstrated poor anti-proliferative effects in all cancer and normal cell-types, with IC_50_ values ranging between 4.74 to >25 µM (Table 1). In contrast, Dp44mT showed selective and potent anti-cancer activity with IC_50_ values of 0.002–0.04 µM, but was markedly less effective in mortal NHDF cells (IC_50_: 15.38 µM; Table 1).

Considering the development of new therapeutics against cancer, it is notable that a lack of expression of the tumor suppressor protein, p53, in some tumors aids the progression of neoplastic cells and promotes resistance against chemotherapeutics [65], [66]. Hence, the use of agents that are active against both wild-type and null p53 tumors are vital, particularly considering the high prevalence of p53 mutations in advanced cancers [42], [67]. Thus, we initially examined the anti-proliferative activity of the novel TSC series **1–6** against human HCT116 p53^+/+^ and HCT116 p53^−/−^ colon cancer cells (Table 1). Significantly, the anti-proliferative effects of most of the novel TSCs showed the same general pattern of activity against HCT116 p53^+/+^ and HCT116 p53^−/−^ cells, irrespective of p53 status (Table 1). One notable exception in the activity between these two cell-types was observed for compound 3e, which showed a 100-fold decrease in anti-proliferative efficacy when comparing HCT116 p53^+/+^ (IC_50_: 0.05 µM) and HCT116 p53^−/−^ cells (IC_50_: 5 µM; Table 1). However, overall, the anti-proliferative activity of the TSCs was generally independent of p53 status (Table 1), as shown for other compounds in this class [16], [42]. Hence, this is an important property of the TSCs that could explain their marked activity that has been observed with related agents across a variety of cancer cell-types [42].

Of all the series of TSCs synthesized in this study, those analogs derived from di-2-pyridyl (**1a–e**) demonstrated the most potent anti-proliferative activity in HCT116 p53^+/+^ and p53^−/−^ cells, resulting in IC_50_ values ranging from 0.0008–0.04 µM (Table 1). While the analogs derived from quinolin-2-yl (**2a–f**) and 8-hydroxyquinolin-2-yl (**3a–f**) demonstrated moderate anti-proliferative effects (IC_50_: 0.026–3.55 and 0.004–9.25 µM, respectively). In contrast, those chelators derived from the 7-hydroxyquinolin-8-yl (**4a–f**), quinoxalin-2-yl (**5a–f**) and salicylic (**6a–f**) moieties showed poor anti-cancer activity (IC_50_: 1.02−>25 µM) in HCT116 p53^+/+^ and p53^−/−^ cells (Table 1).

A similar general trend in the anti-proliferative activity of the novel TSCs to those observed in HCT116 p53^+/+^ and p53^−/−^ cells was also observed in Raji, HeLa and SK-N-MC cells (Table 1). Indeed, those chelators containing the di-2-pyridyl moiety (**1a–e**) were the most potent anti-cancer (IC_50_: 0.0003–2.21 µM) analogs examined. Similarly to the HCT116 data, compounds from series **2** generally displayed moderate anti-proliferative activity (IC_50_: 0.04–5.14 µM) in Raji, HeLa and SK-N-MC cells, while series **3**, **4**, **5** and **6** generally showed moderate to poor anti-proliferative effects (Table 1).

Only a weak to moderate correlation (*R* ^2^  =  0.01–0.6) was evident between the anti-cancer activity of series **1–6** and their log *P*_calc_ values, suggesting that other factors besides their ability to transverse the cellular membrane by passive diffusion were critical in their anti-proliferative effects.

### Anti-Proliferative Activity of the Novel Thiosemicarbazones Against Normal, Mortal Cells

Importantly, for an agent to be useful as an anti-cancer drug, it must show preferential anti-proliferative activity against tumor cells over normal, mortal cell-types. Hence, the selectivity of the novel TSCs was examined in mortal cells, namely NHDF cells. Of all the analogs examined, **1b**, **1d**, **2b**, **2f** and **3c** showed the greatest anti-proliferative activity in the majority of cancer cell-types (Table 1). Thus, to examine the selectivity of the 5 best TSCs identified above, namely **1b**, **1d**, **2b**, **2f** and **3c**, a “therapeutic index” was calculated by dividing the NHDF cell IC_50_ by the IC_50_ of the neoplastic HCT116 p53^+/+^ or HCT116 p53^−/−^ cell-types (Table 2). Notably, the selectivity index of Dp44mT comparing NHDFs to HCT116 p53^+/+^ or HCT116 p53^−/−^ cell-types was marked, being 7690 and 3076, respectively.

**Table 2:** The selectivity of Dp44mT relative to the 5 most potent anti-cancer TSCs, namely **1b**, **1d**, **2b**, **2f** and **3c**, was examined by calculating their “therapeutic indices”.

	Therapeutic index	
Chelator	NHDF *vs.* HCT116 p53^+/+^	NHDF *vs.* HCT116 p53^−/−^
**Dp44mT**	7690	3076
**1b**	2	2
**1d**	80	18
**2b**	0.8	0.4
**2f**	0.6	0.4
**3c**	2650	624

The latter was calculated by dividing the NHDF cell IC_50_ by the IC_50_ of the neoplastic HCT116 p53^+/+^ or HCT116 p53^−/−^ cell-types.

Of the 5 most potent anti-cancer TSCs described herein (Table 1), the greatest therapeutic indices were identified for **1d** and **3c** and were 18–2650 (Table 2). This high selectivity against the cancer cells was due to the fact that both **1d** and **3c** showed potent anti-cancer activity (IC_50_: 0.002–0.017 µM) in HCT116 cells, but their cytotoxicity was greatly reduced in NHDF cells (IC_50_: 0.16–10.6 µM). In contrast, analogs **1b**, **2b** and **2f** demonstrated poor selectivity with IC_50_ values of 0.0008–0.09 µM in HCT116 cells and 0.0017–0.03 µM in mortal NHDF cells (Table 1). In fact, compounds **2b** and **2f** generally showed greater anti-proliferative effects in mortal NHDF cells relative to HCT116 cells, resulting in very low therapeutic indices of 0.4–0.8 (Table 2). Compound **1b** demonstrated limited selectivity and resulted in a therapeutic index of 2 in mortal NHDF cells relative to HCT116 cells. Hence, of all the analogs examined, compounds **1d** and **3c** demonstrated the greatest promise as anti-cancer agents, having both potent and selective anti-proliferative effects.

### Ability of Novel Thiosemicarbazones to Mobilize Cellular ^59^Fe

**Figure 6:**
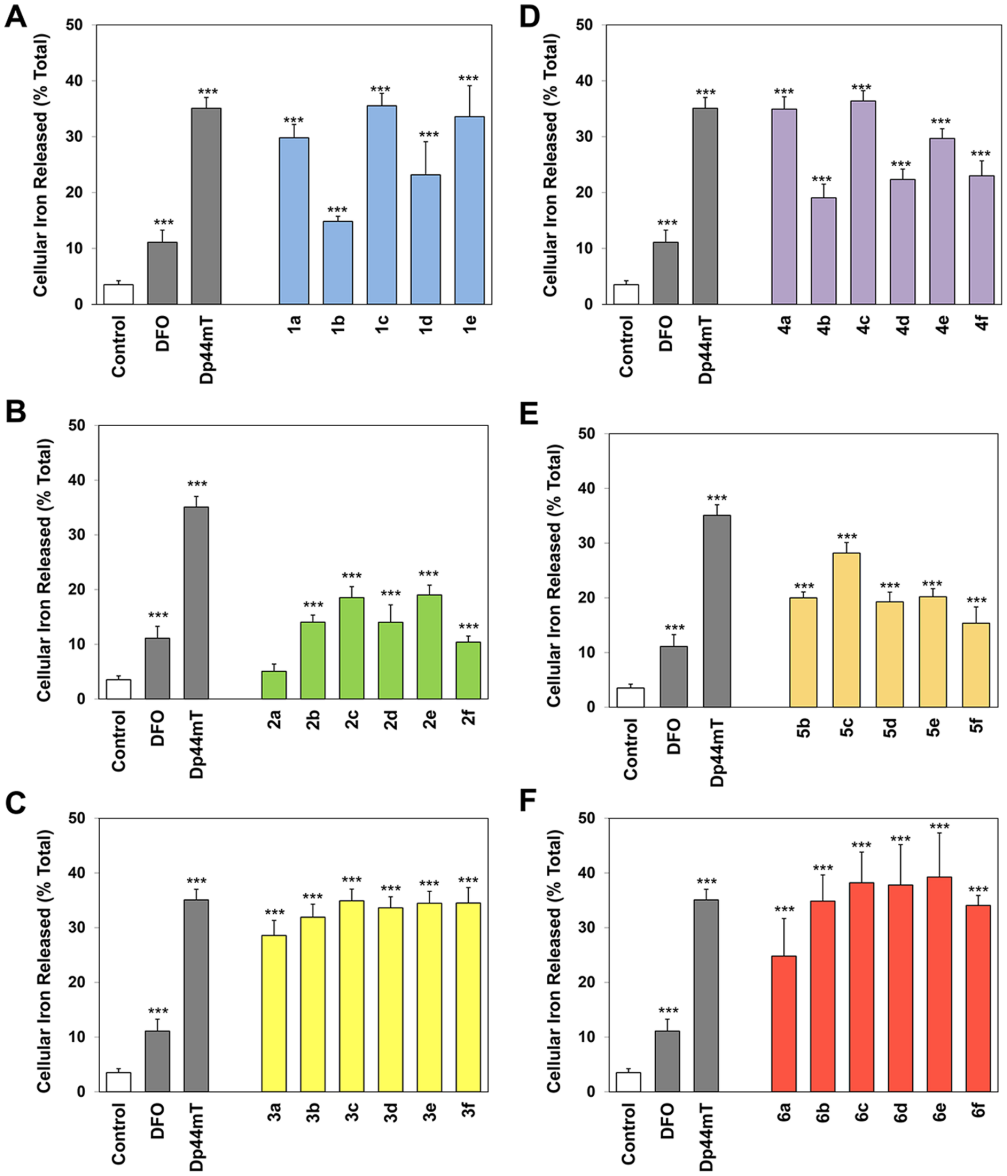
Effect of (A) series 1, (B) series 2, (C) series 3, (D) series 4, (E) series 5 and (F) series 6 chelators, relative to the controls DFO and Dp44mT, on ^59^Fe mobilization from prelabeled SK-N-MC cells. Cells were incubated for 3 h/37°C with ^59^Fe-transferrin (0.75 µM), washed 4 times with ice-cold PBS and then reincubated for 3 h/37°C in the presence or absence of the chelators (25 µM). Release of ^59^Fe was then assessed using a γ-scintillation counter. Results are mean ±SD (3 experiments).

As the ability of chelators to bind cellular iron can play a role in their anti-proliferative effects [50], we examined the ability of these novel ligands (25 µM) to mobilize cellular ^59^Fe from prelabeled SK-N-MC cells (Fig. 6). The chelator-mediated release of intracellular ^59^Fe was compared to the positive controls, DFO and Dp44mT (Fig. 6), as their ability to mobilize ^59^Fe has been extensively characterized in these cells [10], [17], [18], [52], [53]. As previously observed [10], [17], [18], [52], [53], the control medium alone resulted in the release of very little ^59^Fe, namely 4±1% of cellular ^59^Fe (Fig. 6). The chelators, DFO and Dp44mT, significantly (*p*<0.001) increased the mobilization of cellular ^59^Fe to 11±2 and 35±2%, respectively, relative to the control medium (Fig. 6).

Compounds of series **1**, **3**, **4** and **6** generally displayed high efficacy at mobilizing cellular ^59^Fe (Fig. 6). In particular, the ligands, **1c**, **1e**, **3b–3f**, **4a**, **4c**, and **6b–6f** all demonstrated comparable ^59^Fe mobilization efficacy to that of Dp44mT, resulting in the efflux of 31–39% of cellular ^59^Fe. Apart from **1b**, all ligands of series **1**, **3**, **4** and **6** were significantly (*p*<0.001) more effective than DFO in mediating the release of cellular ^59^Fe. Interestingly, ligands of series **1** and **4** demonstrated a similar pattern in terms of their ability to mobilize cellular ^59^Fe (Fig. 6A,D). In both series **1** and **4**, the ligands containing the more hydrophilic fragments, **a**, **c** and **e**, showed increased efficacy at mobilizing cellular ^59^Fe relative to those containing the more lipophilic fragments **b**, **d** and **f** (Fig. 6A,D). Of all the ligands examined, chelators of series **3** and **6** demonstrated the greatest efficacy as ^59^Fe mobilization agents, mediating the release of 25–39% of cellular ^59^Fe (Fig. 6C,F).

In contrast, the series based on quinolin-2-yl (**2**) and quinoxalin-2-yl (**5**) generally demonstrated poor ability to mobilize cellular ^59^Fe and resulted in the release of 5–28% of cellular ^59^Fe (Fig. 6B,E). All ligands of series **2** and **5** were significantly (*p*<0.001) less effective than Dp44mT in mediating the release of cellular ^59^Fe. In fact, ligand **2a** demonstrated comparable ^59^Fe mobilization efficacy to that of the control medium (Fig. 6B), while **2b**, **2d**, **2f** and **5f** showed comparable release of ^59^Fe relative to DFO (Fig. 6B,E).

**Figure 7:**
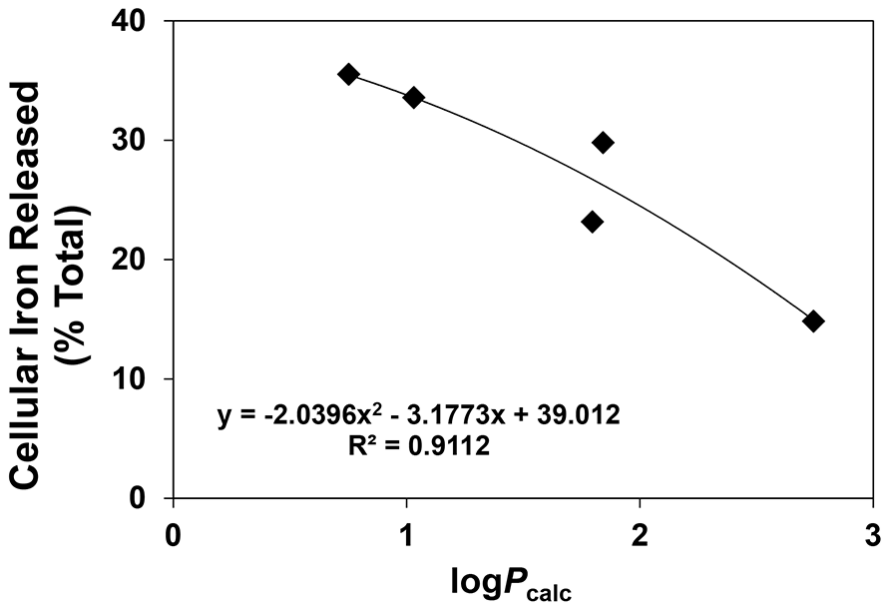
Relationship between the cellular iron released (% total) and lipophilicity (log*P*_calc_) of series 1 chelators using SK-N-MC neuroepithelioma cells. Lines were fitted in using Microsoft Excel 2010 (Microsoft, Redmond, WA).

Importantly, a positive correlation (*R^2^* = 0.911) between log *P*_calc_ and ^59^Fe mobilization efficacy was observed for ligands of series **1** (Fig. 7), suggesting that their lipophilic/hydrophilic balance played a role in their ability to permeate the cellular membrane to reach intracellular ^59^Fe. Interestingly, the more hydrophobic ligands of series **1** were less effective than their more hydrophilic counterparts, suggesting that the more hydrophobic ligands or their resultant ^59^Fe complexes may be sequestered in cellular membranes, preventing ^59^Fe release. In contrast, a poor to moderate correlation (*R^2^* = 0.38–0.67) between log *P*_calc_ and ^59^Fe mobilization efficacy was evident for series **2–6**. A weak correlation (*R^2^* = 0.37) was observed between the anti-proliferative activity of these novel ligands and their ability to mobilize cellular ^59^Fe in SK-N-MC cells, indicating that other factors besides their ability to induce ^59^Fe mobilization were responsible for their anti-cancer effects.

### Ability of Novel Thiosemicarbazones to Inhibit the Cellular Uptake of ^59^Fe from ^59^Fe_2_-Tf

**Figure 8:**
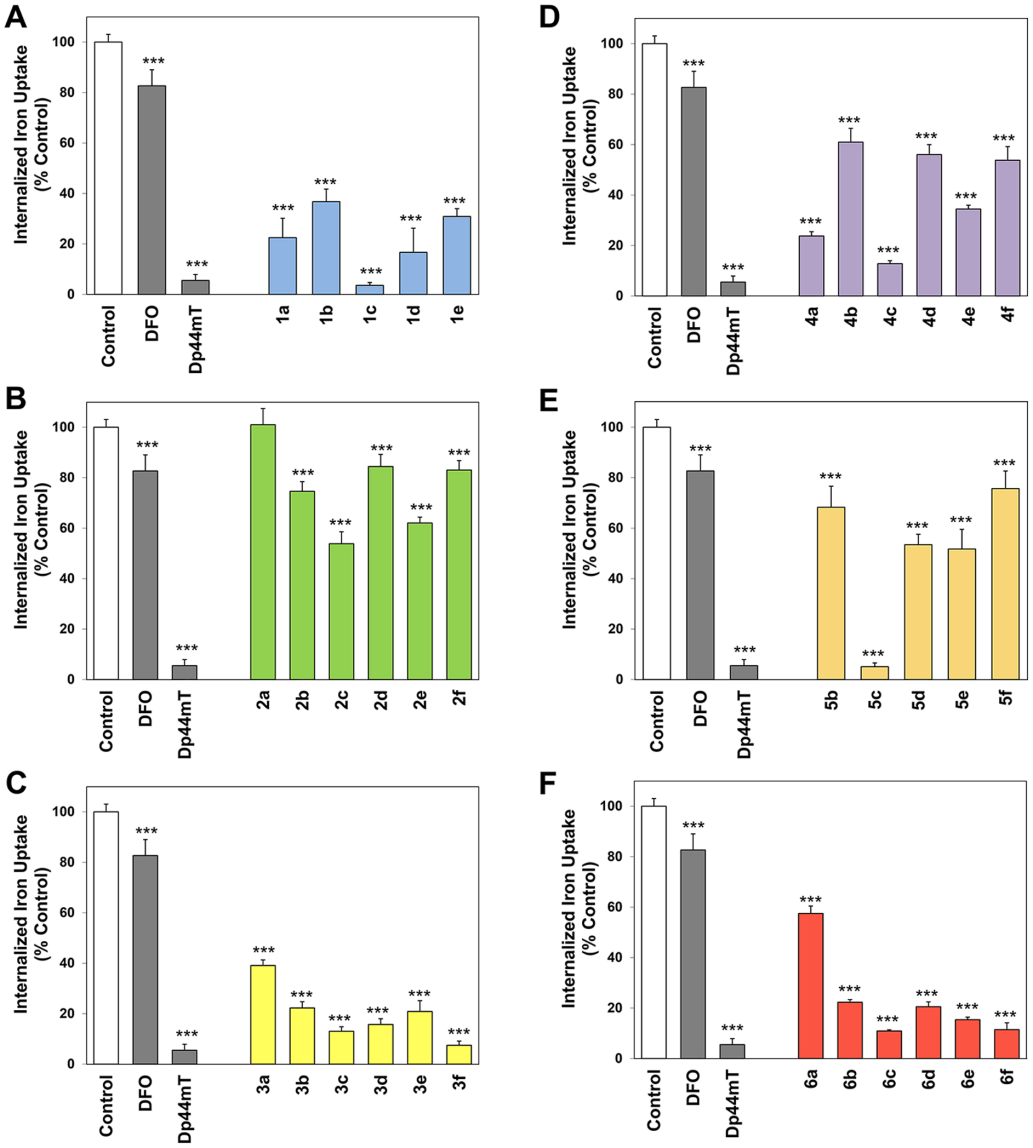
Effect of (A) series 1, (B) series 2, (C) series 3, (D) series 4, (E) series 5 and (F) series 6 chelators, relative to the controls DFO and Dp44mT, on ^59^Fe uptake from ^59^Fe-transferrin by SK-N-MC cells. Cells were incubated for 3 h/37°C with ^59^Fe-transferrin (0.75 µM) in the presence or absence of the chelators (25 µM). At the end of this incubation, cells were washed 4 times with ice-cold PBS. Internalization of ^59^Fe was assessed by incubation for 30 min/4°C with the protease, Pronase (1 mg/mL). Cellular ^59^Fe was then assessed using a γ-scintillation counter. Results are mean ±SD (3 experiments).

The anti-proliferative activity and iron chelation efficacy of a ligand is dependent not only on its ability to mobilize cellular iron, but also on its ability to prevent the cellular uptake of iron from Fe_2_-Tf [50]. Thus, the ability of the novel ligands (25 µM) to inhibit the cellular uptake of ^59^Fe from ^59^Fe_2_-Tf was assessed in SK-N-MC cells (Fig. 8). As utilized in the ^59^Fe efflux experiments, the chelators, DFO and Dp44mT, were included as positive controls as their ability to inhibit ^59^Fe from ^59^Fe_2_-Tf has been extensively assessed [10], [14], [17], [18], [50], [52], [53]. The results were expressed as a percentage of the ^59^Fe uptake observed by cells incubated with ^59^Fe_2_-Tf in control medium (*i.e.*, in the absence of chelator; Fig. 8).

**Figure 9:**
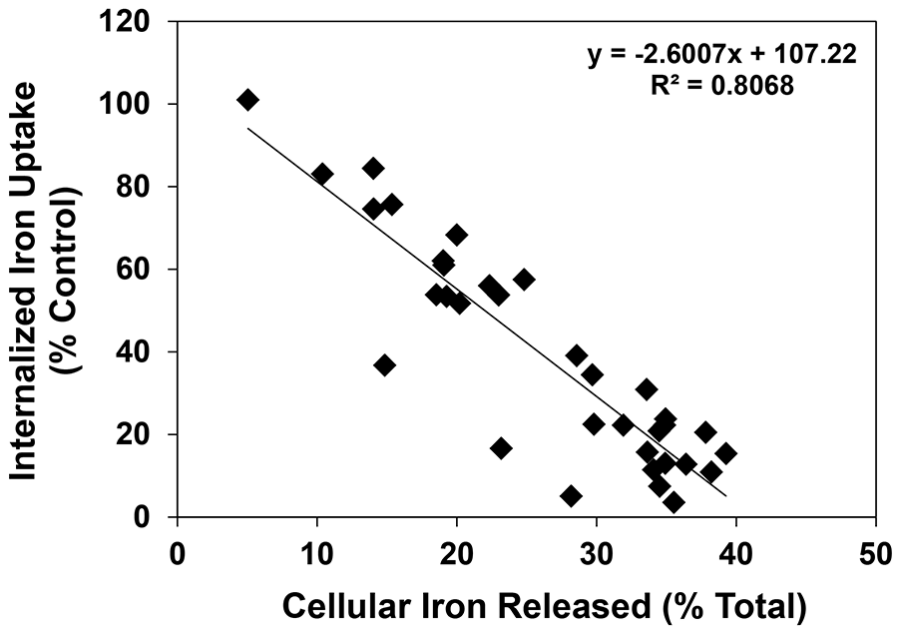
Relationship between the internalized iron uptake (% control) and cellular iron released (% total) of series 1–6 using SK-N-MC neuroepithelioma cells. Lines were fitted in using Microsoft Excel 2010 (Microsoft, Redmond, WA).

As shown previously [10], [52], DFO was able to significantly (*p*<0.001) inhibit ^59^Fe uptake from ^59^Fe_2_-Tf to 83% of that found for the control (Fig. 8), while Dp44mT significantly (*p*<0.001) and markedly prevented the uptake of ^59^Fe, reducing it to 6% of the control (Fig. 8). In general, those analogs that displayed high efficacy at mobilizing cellular ^59^Fe (Fig. 6) also potently inhibited the cellular uptake of ^59^Fe from ^59^Fe_2_-Tf (Fig. 8), with a linear correlation (*R^2^* = 0.81) evident between these 2 factors (Fig. 9). Generally, the ligands of series **1**, **3**, **4** and **6** potently inhibited the uptake of ^59^Fe from ^59^Fe_2_-Tf and were significantly (*p*<0.001) more effective than DFO (Fig. 8A,C,D,F). In fact, the ligands, **1c**, **3f**, **5c**, **6c** and **6f** demonstrated comparable inhibition of cellular ^59^Fe uptake to that of Dp44mT, resulting in 3.6–11.5% of cellular ^59^Fe uptake relative to the control (Fig. 8). Interestingly, those ligands containing the more hydrophilic fragment, **c**, were generally the most effective chelators of each series (Fig. 8).

Similarly to the ^59^Fe efflux experiments, those ligands based on the quinolin-2-yl (**2**) and quinoxalin-2-yl (**5**) moieties generally showed poor ability to prevent the uptake of ^59^Fe from ^59^Fe_2_-Tf (Fig. 8B,E). In fact, compound **2a** showed comparable ability to inhibit ^59^Fe uptake to the control medium, while compounds **2b**, **2d**, **2f** and **5f** demonstrated comparable inhibition of ^59^Fe uptake to that of DFO (Fig. 8B,E). All analogs of series **2** and **5**, except **5c**, were significantly (*p*<0.001) less effective than Dp44mT at inhibiting cellular ^59^Fe uptake.

No strong correlation (*R^2^* = 0.20–0.21) was observed between the ability of the ligands to prevent cellular ^59^Fe uptake and either log *P*_calc_ or anti-proliferative activity in SK-N-MC cells, suggesting other factors besides inhibition of ^59^Fe uptake played a role in their anti-cancer activity.

### Ability of the Iron Complexes of Novel Thiosemicarbazones to Mediate the Oxidation of Ascorbate

**Figure 10:**
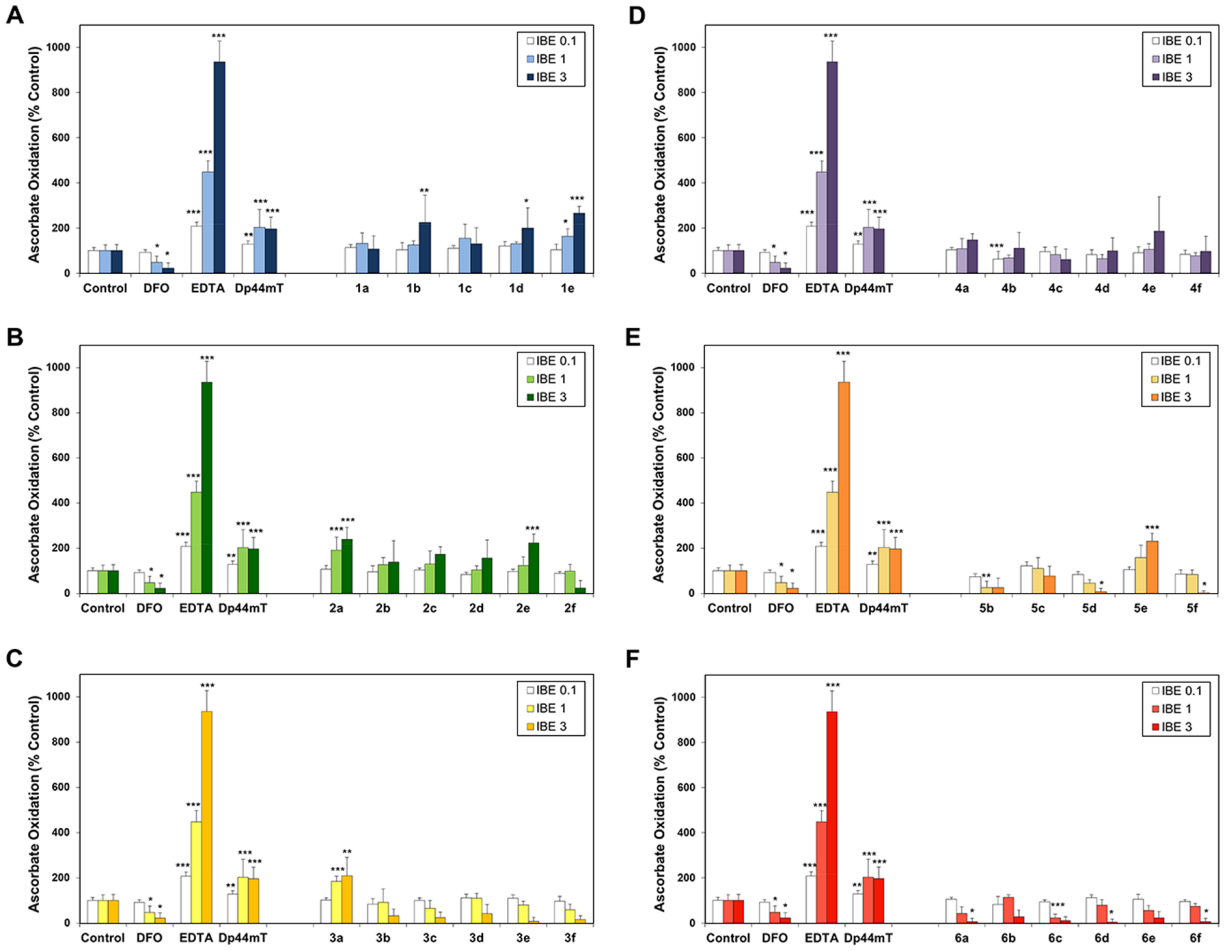
Effect of the iron complexes of (A) series 1, (B) series 2, (C) series 3, (D) series 4, (E) series 5 and (F) series 6 chelators, relative to DFO, Dp44mT and EDTA, on ascorbate oxidation. Chelators at iron-binding equivalent (IBE) ratios of 0.1, 1, and 3 were incubated in the presence of Fe^III^ (10 µM) and ascorbate (100 µM). The UV-Vis absorbance at 265 nm was recorded after 10 and 40 min, and the difference between the time points was calculated. Results are mean ±SD (3 experiments).

The ability of the iron complexes of the novel TSCs to catalyze the oxidation of the physiological substrate, ascorbate, was important to assess as redox cycling may play a critical role in their anti-proliferative activity [10], [15], [18], [52], [53]. Thus, the oxidation of ascorbate mediated by the iron complexes of series **1–6** were examined in comparison to the iron complexes of the control compounds, DFO, EDTA and Dp44mT, as the ability of the latter to oxidize ascorbate is well characterized [10], [15], [52], [53], [68]. The results were expressed as a percentage of the control (no chelator) at iron-binding equivalents (IBEs) of 0.1 (excess “free” iron), 1 (iron-chelator complexes with a fully filled coordination sphere), or 3 (excess free chelator), due to the different denticity of the chelators examined (Fig. 10).

As previously observed, the redox-inactive iron complex of DFO resulted in very limited ascorbate oxidation (Fig. 10) [15], [68], with the ligand acting in a protective manner, significantly (*p*<0.05) inhibiting ascorbate oxidation at IBEs of 1 and 3 to 47% and 23% of the control, respectively (Fig. 10). In contrast, the iron complex of the positive control, EDTA, significantly (*p*<0.001) increased ascorbate oxidation at IBEs of 0.1, 1 and 3 to 208%, 449% and 936%, respectively, in comparison to the control (Fig. 10). This is in agreement with our previous studies that showed its ability to mediate ascorbate oxidation [10], [15], [52], [53]. Additionally, the Dp44mT-iron complex also mediated ascorbate oxidation, significantly (*p*<0.01–0.001) increasing it to 129%, 203% and 196% at IBEs of 0.1, 1 and 3, respectively, relative to the control (Fig. 10), as demonstrated before [10], [15], [52], [53].

The iron complexes of series **1** and **2** generally enhanced ascorbate oxidation, with compounds **1b**, **1d**, **1e**, **2a** and **2e** significantly (*p*<0.05–0.001) increasing ascorbate oxidation to 200–265% of the control at an IBE of 3 (Fig. 10A,B). In fact, all iron chelator complexes of series **1** and **2**, except for **2f**, showed comparable levels of ascorbate oxidation to the Dp44mT-iron complex at an IBE of 3 (Fig 10A,B). These data suggest that the ligands of series **1** and **2** form iron complexes that are redox active.

In contrast to series **1** and **2**, the iron complexes of series **3**, **4**, **5** and **6** generally did not enhance ascorbate oxidation, with the majority acting in a protective manner (Fig. 10C,D,E,F). Although the iron complexes of **3a** and **5e** mediated significantly (*p*<0.01–0.001) increased levels of ascorbate oxidation, the majority of the iron complexes of series **3**, **4**, **5** and **6** resulted in comparable ability to catalyze ascorbate oxidation relative to the control at an IBE of 3. In fact, the iron complexes of **5d**, **5f**, **6a**, **6d** and **6f** significantly (*p*<0.05) decreased ascorbate oxidation relative to the control at an IBE of 3. Additionally, all iron complexes of series **3**, **4**, **5** and **6**, apart from **3a**, **4a**, **4e** and **5e**, mediated comparable ascorbate oxidation to that of DFO at an IBE of 3, resulting in 4–111% of ascorbate oxidation relative to the control (Fig. 10C,D,E,F). Thus, the majority of iron complexes of series **3**, **4**, **5** and **6** acted in a protective manner, suggesting the formation of redox inactive iron complexes.

### Structure-Activity Relationships Linking Anti-Proliferative Activity, Ascorbate Oxidation and Donor Atom Identity

In the current study, series **1** analogs based on the di-2-pyridyl moiety (Fig. 5) generally demonstrated the greatest anti-proliferative activity of all the novel ligands examined (Table 1). Series **1** ligands, that utilize the *N*,*N*,*S* donor atom set, showed high iron chelation efficacy (Figs. 6A,8A) and their iron complexes generally resulted in the increased oxidation of ascorbate (Fig. 10A). These data suggest that the potent anti-proliferative activity of series **1** stemmed from their ability to effectively chelate cellular iron and result in the formation of redox active iron complexes that mediate oxidative damage. This is in agreement with our previous studies on other di-2-pyridyl-based thiosemicarbazones, such as Dp44mT and DpC [12], [15], and di-2-pyridyl-based thiohydrazones [52], which also utilize the *N*,*N*,*S* donor atom set and demonstrate potent anti-cancer activity *via* the formation of redox active complexes. The use of “soft” donor atoms, such as nitrogen and sulfur, play a critical role in facilitating reversible Fe^III/II^ redox cycling reactions that are important for potent anti-cancer effects [12], [15], [52].

The ligands of series **2**, based on the quinolin-2-yl moiety (Fig. 5), also utilized the *N*,*N*,*S* donor atom set and their resultant iron complexes generally increased levels of ascorbate oxidation (Fig. 10B). This suggested that, similarly to series **1**, ligands of series **2** may form redox active iron complexes. However, in contrast to series **1**, series **2** compounds demonstrated poor iron chelation efficacy, which may be a contributing factor in the moderate anti-proliferative activity observed for series **2** relative to series **1** (Table 1). This is consistent with our previous studies, in which the presence of the quinolin-2-yl moiety in 2-quinolinecarboxaldehyde isonicotinoyl hydrazones [59] and other quinoline-based thiosemicarbazones [16] were found to have poor iron mobilization efficacy and anti-proliferative effects in SK-N-MC cells. Our current data support these previous findings, suggesting that the quinolin-2-yl moiety confers poor anti-proliferative and iron chelation efficacy when the quinoline nitrogen acts as a donor atom [16], [59].

Series **3** analogs, that differ from series **2** by the addition of a hydroxy group at position 8 of the quinoline ring (Fig. 5), generally demonstrated moderate to poor anti-proliferative activity (Table 1). In contrast to series **2** that showed poor iron mobilization efficacy and the ability to mediate ascorbate oxidation, series **3** ligands displayed high iron chelation efficacy (Figs. 6C,8C). However, their iron complexes generally did not enhance the oxidation of ascorbate (Fig. 10C). In fact, the majority of series **3** iron complexes acted in a protective manner and inhibited ascorbate oxidation (Fig. 10C). It is probable that series **3** ligands can bind iron either in a tridentate manner, using the *N*,*N*,*S* donor atom set similarly to series **2**, or in a bidentate system similar to that of the structurally-related chelator, clioquinol [69] that utilizes the quinoline nitrogen and 8-hydroxy oxygen as donor atoms [70]. Due to the contrasting activity of series **3** relative to series **2** with regards to anti-proliferative effects (Table 1), iron chelation efficacy (Figs. 6&8) and ascorbate oxidation (Fig. 10), this suggests that the series **3** analogs may act as bidentate ligands that utilize the 8-hydroxyl oxygen and quinoline nitrogen as donor atoms. Significantly, the use of the “hard” oxygen donor atom in series **3** may result in the formation of redox-inactive iron complexes that cannot oxidize ascorbate and consequently show poor anti-proliferative activity [1], [52].

Those ligands based on the 7-hydroxyquinolin-8-yl (series **4**) moiety bind iron in an analogous manner to that of 2-hydroxynaphthaldehyde thiosemicarbazone [71] or 2-hydroxy-1-naphthaldehyde thiobenzoyl hydrazone (H_2_NTBH; [52]) and utilize the *O*,*N*,*S* donor atom set [16]. Similarly, those chelators of series **6**, based on the salicylic moiety, also bind iron *via* the *O*,*N*,*S* donor atom set in a similar manner to salicylaldehyde thiobenzoyl hydrazone (H_2_STBH; [52]). Although the series **4** and **6** analogs demonstrated high iron mobilization efficacy (Figs. 6&8), these ligands showed poor anti-proliferative effects (Table 1) and their iron complexes did not mediate or prevented the oxidation of ascorbate (Fig. 10). This is in agreement with our previous studies in which the *O*,*N*,*S*-thiohydrazones, H_2_STBH or H_2_NTBH, mediated high iron chelation efficacy, but showed poor anti-proliferative activity and the inability of their iron complexes to mediate the oxidation of ascorbate [52]. Importantly, these results suggest that iron complexes of series **4** and **6** ligands cannot mediate the formation of ROS, which greatly reduces their anti-proliferative effects and this relates to the inclusion of the hard oxygen donor (Fig. 5).

The analogs based on quinoxalin-2-yl (**5a–f**) utilize the *N*,*N*,*S* donor atoms and differ from series **2** by an additional non-coordinating nitrogen located at position 4 of the quinoline moiety (Fig. 5). This structural modification resulted in a large decrease in the anti-proliferative activity of series **5** relative to series **2** (Table 1), although both series showed poor iron mobilization efficacy (Figs. 6&8). Importantly, the iron complexes of series **5** acted in a protective manner and inhibited the oxidation of ascorbate, while iron complexes of series **2** were able to promote ascorbate oxidation (Fig. 10). This further highlights the critical role of the formation of redox active iron complexes in the anti-proliferative activity of novel thiosemicarbazones [10], [15], [21], [52]. Significantly, our previous studies on methyl pyrazinylketone isonicotinoyl hydrazone (MPIH) analogs demonstrated that the incorporation of a second, non-coordinating, electron-withdrawing nitrogen in the aromatic ring played a major role in the formation of redox-inactive iron complexes that prevented the formation of ROS [53], [72]. Similarly to series **5**, the iron complexes of the MPIH series prevented the oxidation of ascorbate [53]. Thus, the replacement of the quinoline moiety of series **2** with the quinoxaline group of series **5** resulted in decreased anti-proliferative activity due to the formation of redox-inactive iron complexes.

## Conclusions

In the current study, several novel classes of TSCs were designed that retained the appropriate MW and log *P*_calc_ values to show promise as drug candidates for pharmaceutical development. A combination of retro-fragments that appear in other TSC precursors were utilized and di-substitution at the terminal N4 atom was preserved through the incorporation of an N4-based piperazine or morpholine ring. The selectivity and anti-cancer activity of the novel TSCs were examined in a variety of cancer cell-types. In particular, of all the compounds examined, **1d** and **3c** demonstrated the greatest promise as anti-cancer agents with both potent and selective anti-proliferative activity (Tables 1 and 2).

Structure-activity relationship studies revealed that the combination of the donor atoms used, rather than the identity of fragments **a–f**, played a crucial role in their anti-cancer activity. Indeed, the chelators that utilized “soft” donor atoms, such as nitrogen and sulfur, formed redox active iron complexes capable of mediating ascorbate oxidation. This further highlights the important role of ROS generation in mediating potent anti-cancer effects. Significantly, this study identified potent and selective anti-cancer chelators that warrant further *in vivo* examination.

## Supporting Information

File S1 **This file reports the chemical characterization of thiosemicarbazides and thiosemicarbazones, X-ray data for selected thiosemicarbazones and thiosemicarbazides, HPLC purity data, isosbestic curves and anti-proliferative activity color maps. Figure S1,** The crystal structure of 4-ethylpiperazine-1-carbothiohydrazide (**a**). **Figure S2**, The crystal structure of *Z*-*N*′-(di(pyridin-2-yl)methylene)-4-(pyridin-2-yl)piperazine-1-carbothiohydrazide (**1d**). **Figure S3**, The absorbance spectrum of **2f** and its Fe^3+^ complexes prepared *in situ* to obtain 1∶1, 2∶1, 4∶1, 5∶1, and 10∶1 ligand:Fe ratios. **Figure S4**, Color maps of the anti-proliferative activity of series **1–6** in several tumor cell-types and normal human dermal fibroblast (NHDF) cells. Red represents the thiosemicarbazones with the greatest anti-proliferative activity (IC50: <1 µM), yellow represents the thiosemicarbazones with moderate activity (IC50: 1–6.25 µM) and grey represents those analogs with poor anti-proliferative effects (IC50>6.25 µM). **Table S1**, Crystal data of **a** and **1d**. **Table S2**, HPLC purity data for all chelators of series **1–6**.
(PDF)
