# Supplementary material for: Exploring the Anti-Cancer Activity of Novel Thiosemicarbazones Generated through the Combination of Retro-Fragments: Dissection of Critical Structure-Activity Relationships
Source: PLoS One. 2014 Oct 16;9(10):e110291. doi: 10.1371/journal.pone.0110291 (PMC4199632; doi:10.1371/journal.pone.0110291)
Supplement: File S1 — This file reports the chemical characterization of thiosemicarbazides and thiosemicarbazones, X-ray data for selected thiosemicarbazones and thiosemicarbazides, HPLC purity data, isosbestic curves and anti-proliferative activity color maps. Figure S1, The crystal structure of 4-ethylpiperazine-1-carbothiohydrazide (a). Figure S2, The crystal structure of Z-N′-(di(pyridin-2-yl)methylene)-4-(pyridin-2-yl)piperazine-1-carbothiohydrazide (1d). Figure S3, The absorbance spectrum of 2f and its Fe3+ complexes prepared in situ to obtain 1∶1, 2∶1, 4∶1, 5∶1, and 10∶1 ligand:Fe ratios. Figure S4, Color maps of the anti-proliferative activity of series 1–6 in several tumor cell-types and normal human dermal fibroblast (NHDF) cells. Red represents the thiosemicarbazones with the greatest anti-proliferative activity (IC50: <1 µM), yellow represents the thiosemicarbazones with moderate activity (IC50: 1–6.25 µM) and grey represents those analogs with poor anti-proliferative effects (IC50>6.25 µM). Table S1, Crystal data of a and 1d. Table S2, HPLC purity data for all chelators of series 1–6. (PDF) [file pone.0110291.s001.pdf]

Exploring the anti-cancer activity of novel thiosemicarbazones generated through the combination of retro-fragments: Dissection of critical structure-activity relationships

Maciej Serda[1†], Danuta S. Kalinowski[2†], Nathalie Rasko[2], Eliška Potůčková[3], Anna Mrozek-Wilczkiewicz[1,4], Robert Musiol[1], Jan G. Małecki[1], Mieczysław Sajewicz[1], Alicja Ratuszna[4], Angelika Muchowicz[5], Jakub Gołąb[5,6], Tomáš Šimůnek[3], Des R. Richardson[2*], Jaroslaw Polanski[1*]

[1]Institute of Chemistry, University of Silesia, Katowice, Silesia, Poland; [2]Department of Pathology and Bosch Institute, University of Sydney, Sydney, New South Wales, Australia; [3]Department of Biochemical Sciences, Charles University in Prague, Faculty of Pharmacy in Hradec Králové, Hradec Králové, Czech Republic; [4]A.Chełkowski Institute of Physics and Silesian Interdisciplinary Centre for Education and Research, University of Silesia, Katowice, Silesia, Poland; [5]Department of Immunology, Medical University of Warsaw, Warsaw, Mazovia, Poland; [6]Institute of Physical Chemistry, Polish Academy of Sciences, Warsaw, Mazovia, Poland.

[†] M.S. and D.S.K. contributed as equal first authors.

***Authors for Correspondence:** Dr. J. Polanski e-mail: polanski@us.edu.pl and Dr. D.R. Richardson email: d.richardson@med.usyd.edu.au

S1 Chemistry

- **S1.1 Chemical characterization of thiosemicarbazides and thiosemicarbazones**
- **S1.2 X-ray data for selected thiosemicarbazones and thiosemicarbazides**
- **S1.3 HPLC purity data**
- **S1.4 Isosbestic curves**

S2 Anti-proliferative activity color maps

S3 References

S1 Chemistry

General reagents were purchased from Sigma-Aldrich (St. Louis, MO, USA), ACROS Organics (Belgium) or Princeton Chemicals Ltd (Luton, Bedfordshire, UK). Silica gel 60 (0.040-0.063 mm; Merck, Darmstadt, Germany) was used for column chromatography. Thin layer chromatography (TLC) was performed on alumina-backed silica gel 40 F_{254} plates (Merck). The plates were illuminated under UV (254 nm) and evaluated in iodine vapor. The melting points were determined on Optimelt MPA100 instrument (SRS, USA) and are uncorrected. Syntheses were performed on a CEM-DISCOVERY microwave reactor (CEM Corporation, Matthews, NC, USA) with temperature and pressure control. High resolution-mass spectrometry (HRMS) analysis was performed for all new compounds on a Finnigan MAT95 spectrometer (Thermo Fisher Scientifc, Bremen, GmbH) or on Mariner ESI-TOF spectrometer (Applied Biosystems, USA). The purity of all novel compounds was assessed using a Gynkotek HPLC Modular System equipped with a DAAD UVD340U detector at 250 nm.

All ^{1}H NMR spectra were recorded on a Bruker AM-400 spectrometer (399.95 MHz for ^{1}H; 99.99 MHz for ^{13}C; BrukerBioSpin Corp., Germany). Chemical shifts are reported in ppm against the internal standard, $Si(CH_3)_4$. Easily exchangeable signals were omitted when diffuse.

LogP_{calc} values were calculated using ChemDraw 12 (Perkin-Elmer, Waltham, MA, USA) by using Crippen's fragmentation [1], Viswanadhan's fragmentation [2] and Broto's method [3] and then calculating the average logP_{calc}.

S1.1 Chemical Characterization of Thiosemicarbazides and Thiosemicarbazones

The thiosemicarbazides (**a**-**f**) and their thiosemicarbazones (Series **1**-**6**) were prepared according to Schemes 1-3. The characterization of all novel products is described below.

4-Ethylpiperazine-1-carbothiohydrazide **(a)**

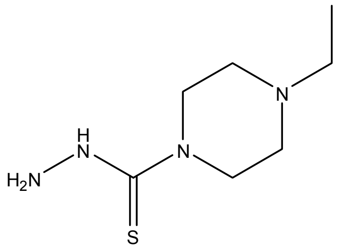

Yield: 47%. Purity: 98.67%. ^{1}H-NMR (d_6-DMSO, 400 MHz, ppm): 3.71-3.68 (m, 4H, CH_2), 2.35-2.29 (m, 6H, CH_2), 1.00 (t, 3H, J= 7.2 Hz). ^{13}C-NMR (d_6-DMSO, 100 MHz, ppm): 183.0, 52.45, 51.86, 47.75, 12.35. MP: 137-138°C (ethanol). HRMS-ESI-TOF: 189.1171 [M + H]$^+$ ($C_7H_{17}N_4S$; Exact Mass: 189.1174). Log P_{calc}: 0.57.

4-Phenylpiperazine-1-carbothiohydrazide **(b)**

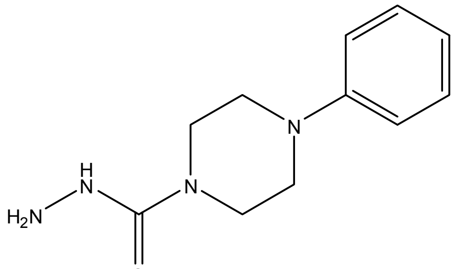

Yield: 58%. MP: 161-162°C (ethanol) [176-177°C (dioxane) [4]]. Log P_{calc}: 1.472.

Morpholine-4-carbothiohydrazide **(c)**

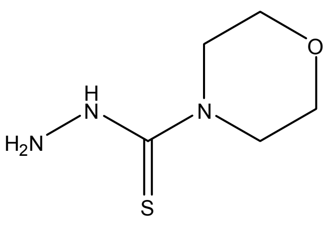

Yield: 76%. MP: 170-171°C (ethanol) [170-171°C; [5]] LogP_{calc}: -0.52.

4-(Pyridin-2-yl)piperazine-1-carbothiohydrazide **(d)**

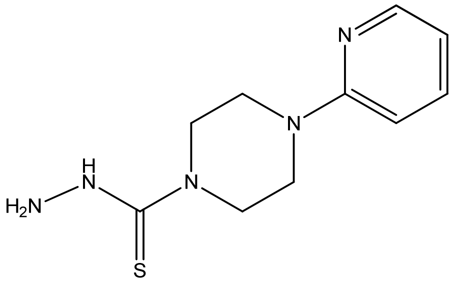

Yield: 95%. MP: 177-178°C (ethanol) [179-180°C; [4]]. Log P_{calc}: 0.524.

4-(Pyrazin-2-yl)piperazine-1-carbothiohydrazide **(e)**

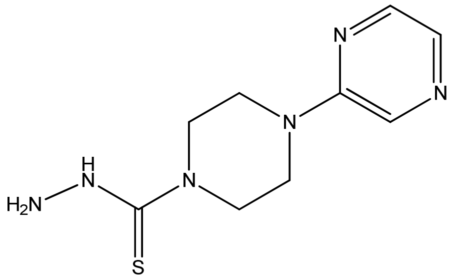

Yield: 95%. Purity: 96.98% (250 nm). ^{1}H-NMR (d_6-DMSO, 400 MHz, ppm): 9.17 (bs, 1H, NH), 8.31 (bs, 1H, pyrazine), 8.09 (bs, 1H, pyrazine), 7.86 (bs, 1H, pyrazine), 4.83 (bs, 2H, NH), 3.89 (m, 4H, CH_2), 3.61 (m, 4H, CH_2). ^{13}C-NMR (d_6-DMSO, 100 MHz, ppm): 183.0, 154.7, 141.9, 133.1, 131.7, 47.0, 43.7. MP: 167-168°C (ethanol). HRMS-ESI-TOF: 239.1080 $[M + H]^+$ ($C_9H_{15}N_6S$; Exact Mass: 239.1079). Log P_{calc}: -0.24.

N-Cyclohexyl-N-methylhydrazinecarbothioamide **(f)**

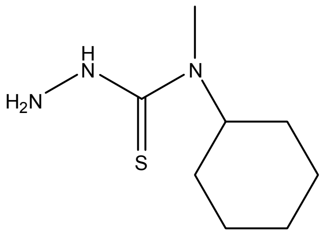

Yield: 66%. MP:138-139°C (ethanol) [140°C; [6]] Log P_{calc}: 1.733.

***(Z)*-*N'*-(Di(pyridin-2-yl)methylene)-4-ethylpiperazine-1-carbothiohydrazide (1a)**

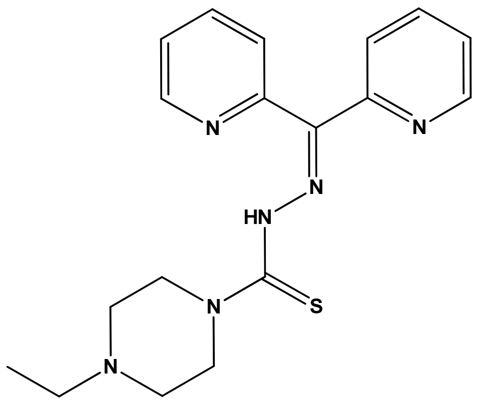

Yield: 62%. Purity: 97.32%. ^{1}H-NMR (d_6-DMSO, 400 MHz, ppm): 14.47 (bs, 1H, NH), 8.85 (d, 1H, *J*= 3,6 Hz), 8.60 (d, 1H, *J*= 3.6 Hz), 8.01-7.91 (m, 3H), 7.59 (m, 2H), 7.58 (m, 1H), 4.00 (m, 4H, CH_2, piperazine), 2.50 (m, 6H, CH_2, piperazine), 1.06 (t, 3H, *J*= 6.8 Hz, CH_3). ^{13}C-NMR (d_6-DMSO, 100 MHz, ppm): 180.8, 148.9, 148.3, 138.3, 127.4, 125.1, 124.2, 56.5, 52.4, 51.7, 12.4. MP: 144-145°C. R_f= 0.52 [dichloromethane:ethanol 16:1 (v/v)]. HRMS-EI: 354.1626 ($C_{18}H_{22}N_6S$; Exact Mass: 354.1627). Log P_{calc}: 1.842.

***N'*-(Di(pyridin-2-yl)methylene)-4-phenylpiperazine-1-carbothiohydrazide (1b)**

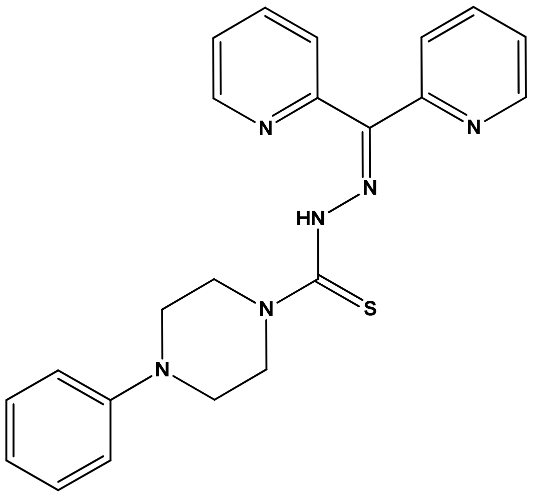

Yield: 78%. Purity: 98.65%. ^{1}H-NMR (d_6-DMSO, 400 MHz, ppm): 14.59 (bs, 1H, NH), 8.89 (d, 1H, *J*= 3.9 Hz), 8.60 (d, 1H, *J*= 4.0 Hz, pyridine), 8.01-7.91 (m, 3H), 7.60 (m, 2H), 7.49 (m, 1H), 7.25 (t, 2H, *J*= 7.7 Hz), 6.96 (d, 2H, *J*= 8.2 Hz), 6.81 (t, 1H, *J*= 7.2 Hz), 4.17 (m, 4H, CH_2, piperazine), 3.34 (m, 4H, CH_2, piperazine). ^{13}C-NMR (d_6-DMSO, 100 MHz,

ppm):180.7, 150.8, 148.7, 148.4, 138.3, 137.7, 129.5, 127.3, 125.1, 119.4, 115.7, 49.6, 48.1. MP: 151-152°C (151-152°C, [7]). R_f= 0.28 [dichloromethane:methanol 40:1 (v/v)]. HRMS-EI: 402.1628 ($C_{22}H_{22}N_6S$; Exact Mass: 402.1627). LogP_{calc}: 2.744.

N'-(Di(pyridin-2-yl)methylene)morpholine-4-carbothiohydrazide (1c)

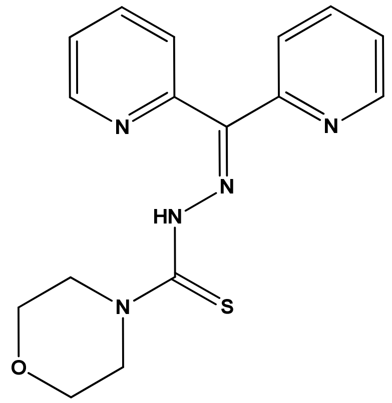

Yield: 78%. Purity: 96.32%. ^{1}H-NMR (d_6-DMSO, 400 MHz, ppm): 14.50 (bs, 1H, NH), 8.85 (d, 1H, J= 3,7 Hz), 8.61 (d, 1H, J= 3.5 Hz), 8.01-7.91 (m, 3H), 7.58 (m, 2H), 7.49 (m, 1H), 4.00 (m, 4H, CH_2); 3.71 (m, 4H, CH_2). ^{13}C-NMR (d_6-DMSO, 100 MHz, ppm):180.2, 148.3, 148.4, 138.7, 127.5, 125.1, 124.0, 68.5, 52.6. MP: 124-125°C. R_f= 0.57 [dichloromethane:ethyl acetate 8:1 (v/v)]. HRMS-EI: 327.1158 ($C_{16}H_{17}N_5OS$; Exact Mass: 327.1154). Log P_{calc}: 0.752.

***(Z)*-N'-(Di(pyridin-2-yl)methylene)-4-(pyridin-2-yl)piperazine-1-carbothiohydrazide (1d)**

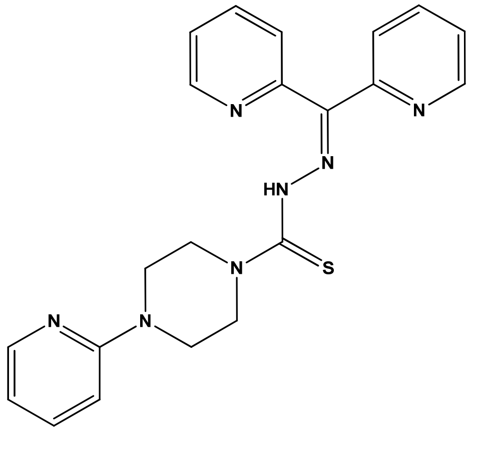

Yield: 74%. Purity: 95.62%. ^{1}H-NMR (d_6-DMSO, 400 MHz, ppm): 14.65 (bs, 1H, NH), 8.90 (d, 1H, J= 5.2 Hz, pyridine), 8.62 (d, 1H, J= 4.4 Hz, pyridine), 8.15 (dd, 1H, J_1= 4.6 Hz, J_2= 1.5 Hz, pyridine), 8.05-7.93 (m, 3H, pyridine), 7.62-7.55 (m, 3H, pyridine), 7.49 (t, 1H, J= 7.2 Hz, pyridine), 6.82 (d, 1H, J= 8.6 Hz, pyridine), 6.68 (dd, 1H, J_1= 6.8 Hz, J_2= 5.2 Hz, pyridine), 4.15 (m, 4H, CH_2, piperazine), 3.72 (m, 4H, CH_2, piperazine). ^{13}C-NMR (d_6-DMSO, 100 MHz, ppm): 180.7, 158.9, 148.9, 148.4, 148.0, 138.3, 137.8, 127.3, 125.1, 124.2, 113.5, 107.3, 49.0, 44.2. MP: 143-144°C. HRMS-ESI-TOF: 404.1652 [M + H]$^+$ ($C_{21}H_{22}N_7S$; Exact Mass: 404.1657). Log P_{calc}: 1.796.

***N'*-(Di(pyridin-2-yl)methylene)-4-(pyrazin-2-yl)piperazine-1-carbothiohydrazide (1e)**

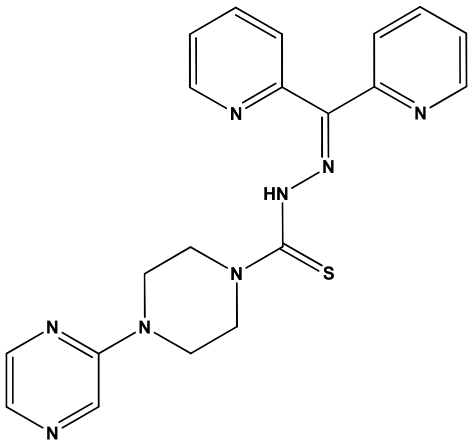

Yield: 66%. Purity: 95.45%. ^{1}H-NMR (d_6-DMSO, 400 MHz, ppm): 14.66 (bs, 1H, NH), 8.89 (d, 1H, J = 4.7 Hz, pyridine), 8.62 (d, 1H, J = 4.0 Hz), 8.31 (bs, 1H, pyrazine), 8.12 (bs, 1H, pyrazine), 8.06 – 7.91 (m, 3H, pyridine), 7.88 (d, 1H, J = 2.2 Hz, pyrazine), 7.61 (t, 2H, J= 6.4 Hz, pyridine), 7.49 (t, 1H, J= 5.6 Hz, pyridine), 4.18 (m, 4H, CH_2, piperazine), 3.80 (m, 4H, CH_2, piperazine). ^{13}C-NMR (d_6-DMSO, 100 MHz, ppm): 180.7, 154.5, 149.4, 148.9, 148.4, 141.9, 138.2, 137.8, 127.3, 125.1, 124.2, 48.8, 43.5. MP: 175-176°C. HRMS-ESI-TOF: 405.1606 [M + H]$^+$ ($C_{20}H_{21}N_8S$; Exact Mass: 405.1610). Log P_{calc}: 1.031.

(*E*)-4-Ethyl-*N'*-(quinolin-2-ylmethylene)piperazine-1-carbothiohydrazide (2a)

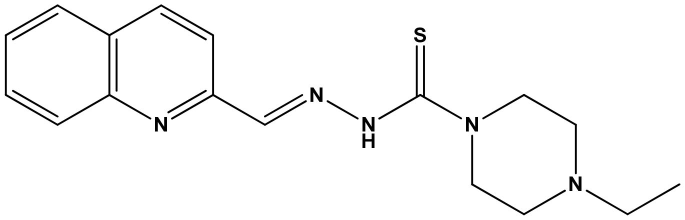

Yield: 78%. Purity: 96.67%. ^{1}H-NMR (d_6-DMSO, 400 MHz, ppm): 8.39 (d, 1H, *J*= 8.7 Hz, quinoline), 8.32 (s, 1H, CH), 8.03-7.98 (m, 3H, quinoline), 7.77 (t, 1H, *J*= 8.4 Hz, quinoline), 7.62 (t, 1H, *J*= 7.8 Hz, quinoline), 3.97 (m, 4H, CH_2, piperazine), 2.51 (m, 6H, CH_2, piperazine, $\underline{CH_2}$-CH_3), 1.04 (t, 3H, *J*= 7.2 Hz, CH_2-$\underline{CH_3}$). ^{13}C-NMR (d_6-DMSO, 100 MHz, ppm): 181.1, 154.3, 147.8, 144.2, 137.1, 130.5, 129.2, 128.5, 128.2, 127.6, 117.7, 52.6, 51.8, 50.5, 12.3. MP: 168-169°C. R_f= 0.48 [dichloromethane:ethanol 10:1 (v/v)]. HRMS-ESI-TOF: 350.1414 $[M + Na]^+$ ($C_{17}H_{21}N_5SNa$; Exact Mass: 350.1415). Log P_{calc}: 2.883.

(*E*)-4-Phenyl-*N'*-(quinolin-2-ylmethylene)piperazine-1-carbothiohydrazide (2b)

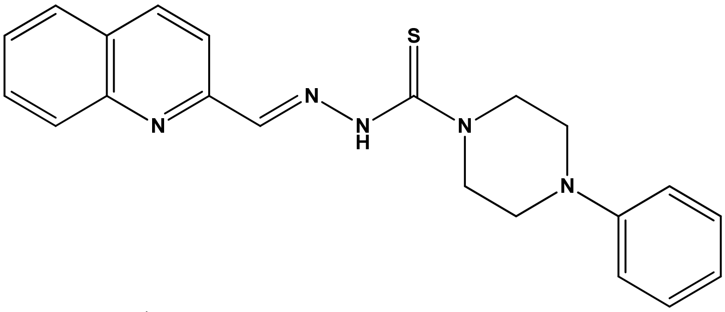

Yield: 81%. Purity: 98.00%. ^{1}H-NMR (d_6-DMSO, 400 MHz, ppm): 11.66 (bs, 1H, NH), 8.39 (d, 1H, *J*= 8.7 Hz, quinoline), 8.35 (s, 1H, CH), 8.03 (d, 2H, *J*= 8.5 Hz, quinoline), 7.99 (d, 1H, *J*= 8.0 Hz, quinoline), 7.79 (t, 1H, *J*= 7.6 Hz, quinoline), 7.62 (t, 1H, *J*= 7.8 Hz, quinoline), 7.25 (t, 2H, *J* = 7.8 Hz, phenyl), 6.99 (d, 2H, *J* = 8.1 Hz, phenyl), 6.82 (t, 1H, *J* = 7.1 Hz, phenyl), 4.14 (m, 4H, CH_2, piperazine), 3.32 (m, 4H, CH_2, piperazine). ^{13}C-NMR (d_6-DMSO, 100 MHz, ppm): 181.2, 154.3, 150.9, 147.9, 144.4, 137.1, 130.5, 129.5, 129.3, 128.5, 128.2, 127.6, 119.6, 117.8, 116.0, 50.3, 48.5. MP: 162-163°C. HRMS-ESI-TOF: 376.1591 $[M + H]^+$ ($C_{21}H_{22}N_5S$; Exact Mass: 376.1596). Log P_{calc}: 3.785.

(*E*)-*N*'-(Quinolin-2-ylmethylene)morpholine-4-carbothiohydrazide (2c)

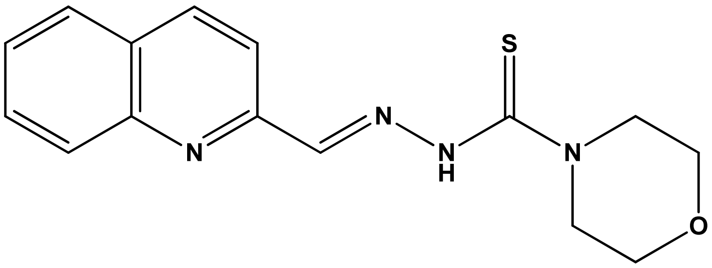

Yield: 85%. Purity: 97.42%. ^{1}H-NMR (d_6-DMSO, 400 MHz, ppm): 11.61 (bs, 1H, NH), 8.38 (d, 1H, J = 8.7 Hz, quinoline), 8.32 (s, 1H, CH), 8.01 (m, 3H, quinoline), 7.80 (t, 1H, J= 8.0 Hz, quinoline), 7.64 (t, 1H, J= 6.4 Hz, quinoline), 3.99 (m, 4H, piperazine), 3.72 (m, 4H, piperazine). ^{13}C-NMR(d_6-DMSO, 100 MHz, ppm):181.4, 154.3, 147.8, 144.4, 137.1, 130.5, 129.3, 128.4, 128.2, 127.6, 117.7, 66.5, 51.2. MP: 145-146°C. HRMS-EI: 300.1034 ($C_{15}H_{16}N_4OS$; Exact Mass: 300.1045). Log P_{calc}: 1.793.

(*E*)-4-(Pyridin-2-yl)-*N*'-(quinolin-2-ylmethylene)piperazine-1-carbothiohydrazide (2d)

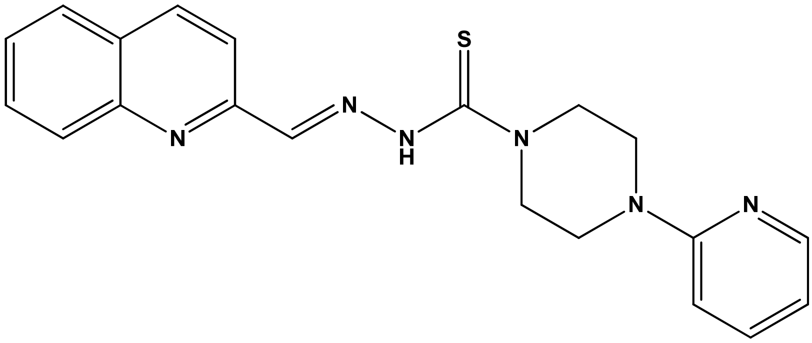

Yield: 69%. Purity: 97.87%. ^{1}H-NMR (d_6-DMSO, 400 MHz, ppm): 11.50 (bs, 1H, NH), 8.38 (m, 2H, quinoline, pyridine), 8.16 (d, 1H, J= 4.0 Hz, pyridine), 8.03 (d, 2H, J= 8.6 Hz, quinoline), 7.98 (d, 1H, J= 8.0 Hz, quinoline), 7.79 (t, 1H, J= 7.2 Hz, quinoline), 7.64-7.56 (m, 2H, quinoline, pyridine), 6.85 (d, 1H, J= 8.8 Hz, pyridine), 6.68 (t, 1H, J= 5.2 Hz, pyridine), 4.12 (m, 4H, piperazine), 3.71 (m, 4H, piperazine). ^{13}C-NMR, (d_6-DMSO, 100 MHz, ppm):181.5, 159.0, 154.3, 148.0, 147.9, 144.5, 138.0, 137.1, 130.4, 129.3, 128.4, 128.2,

127.6, 117.8, 113.6, 107.5, 50.2, 44.7. MP: 133-134°C. HRMS-ESI-TOF: 377.1549 $[M + H]^+$ ($C_{20}H_{21}N_6S$; Exact Mass: 377.1548). Log P_{calc}: 2.837.

(*E*)-4-(Pyrazin-2-yl)-*N*'-(quinolin-2-ylmethylene)piperazine-1-carbothiohydrazide (2e)

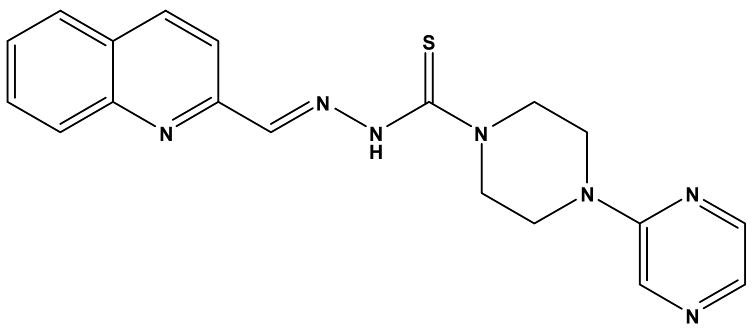

Yield: 96%. Purity: 98.98%. ^{1}H-NMR (d_6-DMSO, 400 MHz, ppm): 11.77 (bs, 1H, NH), 8.39 (d, 1H, *J*= 8.7 Hz), 8.35 (m, 2H, CH, pyrazine), 8.13 (m, 1H), 8.03 (m, 2H), 8.00 (d, 1H, *J*= 7.8 Hz), 7.89 (d, 1H, *J*= 2.4 Hz), 7.79 (t, 1H, *J*= 7.0 Hz), 7.63 (t, 1H, *J*= 7.9 Hz), 4.13 (m, 4H, piperazine), 3.78 (m, 4H, piperazine). ^{13}C-NMR (d_6-DMSO, 100 MHz, ppm):181.3, 154.7, 154.3, 147.9, 144.4, 141.9, 137.1, 133.1, 131.7, 130.5, 129.3, 128.5, 128.2, 127.6, 117.8, 49.9, 44.0. MP: 188-189° C. HRMS-ESI-TOF: 378.1498 $[M + H]^+$ ($C_{19}H_{20}N_7S$; Exact Mass: 378.1501). Log P_{calc}: 2.072.

(*E*)-*N*-Cyclohexyl-*N*-methyl-2-(quinolin-2-ylmethylene)hydrazinecarbothioamide (2f)

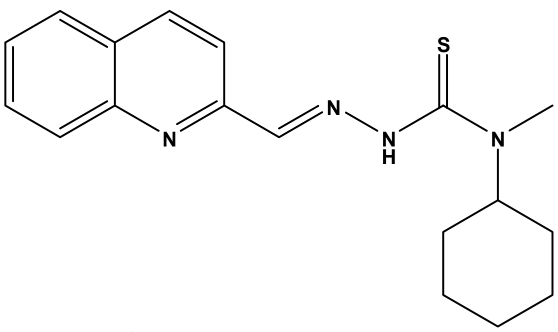

Yield: 58%. Purity: 99.12%. ^{1}H-NMR (d_6-DMSO, 400 MHz, ppm): 11.25 (bs, 1H, NH), 8.39 (m, 2H, CH, quinoline), 8.03-7.98 (m, 3H, quinoline), 7.78 (t, 1H, *J*= 8.0 Hz, quinoline), 7.62 (t, 1H, *J*= 7.6 Hz, quinoline), 4.74 (bs, 1H, C_1-cyclohexyl), 3.14 (s, 3H, CH_3), 1.80 (m, 4H,

cyclohexyl), 1.58 (m, 3H, cyclohexyl), 1.30 (m, 2H, cyclohexyl), 1.15 (t, 1H, *J*= 12.0 Hz, cyclohexyl). ^{13}C-NMR (d_6-DMSO, 100 MHz, ppm):181.1, 154.5, 147.9, 144.2, 137.0, 130.5, 129.2, 128.4, 128.1, 127.5, 117.7, 61.2, 39.8, 34.9, 29.7, 25.8, 25.7, 25.4. MP: 125-126°C. HRMS-EI: 326.1564 ($C_{18}H_{22}N_4S$; Exact Mass: 326.1565). Log P_{calc}: 4.145.

(*Z*)-4-Ethyl-*N'*-((8-hydroxyquinolin-2-yl)methylene)piperazine-1-carbothiohydrazide (3a)

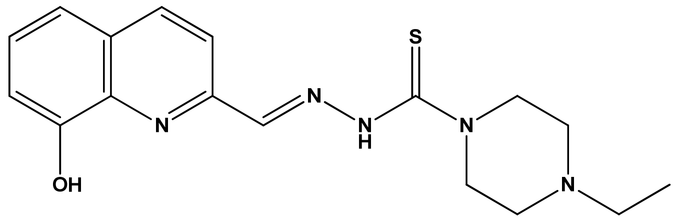

Yield: 74%. Purity: 95.03%. ^{1}H-NMR (d_6-DMSO, 400 MHz, ppm): 15.29 (bs, 1H, NH), 8.33 (s,1H, CH), 8.30 (d, 1H, *J*= 8.4 Hz, quinoline), 7.97 (d, 1H, *J*= 8.4 Hz, quinoline), 7.44 (t, 1H *J* = 7.6 Hz), 7.38 (d, 1H, *J* = 7.2 Hz), 7.11 (d, 1H *J* = 7.4 Hz), 3.95 (m, 4H, piperazine), 2.50 (m, 6H, piperazine, CH_2), 1.04 (t, 3H, *J*= 6.8 Hz). ^{13}C-NMR (d_6-DMSO, 100 MHz, ppm):181.2, 153.8, 152.3, 144.0, 138.6, 136.9, 129.1, 128.5, 118.2, 117.9, 112.6, 52.7, 51.7, 50.5, 12.4. MP: 167-168°C. HRMS-ESI-TOF: 366.1362 $[M + Na]^+$ ($C_{17}H_{21}N_5OSNa$; Exact Mass: 366.1365). Log P_{calc}: 2.931.

(*E*)-*N'*-((8-Hydroxyquinolin-2-yl)methylene)-4-phenylpiperazine-1-carbothio-hydrazide (3b)

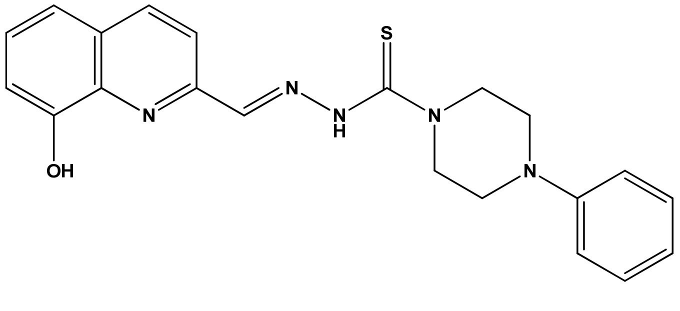

Yield: 67%. Purity: 96.59%. ^{1}H-NMR (d_6-DMSO, 400 MHz, ppm): 11.71 (bs, 1H, NH), 9.83 (bs, 1H, OH), 8.38 (bs, 1H, CH), 8.32 (d, 1H, *J*= 8.7 Hz, quinoline), 8.01 (d, 1H, *J*= 8.7 Hz,

quinoline), 7.44 (m, 1H, quinoline), 7.39 (m, 1H), 7. 25 (t, 2H, *J*= 7.9 Hz, phenyl), 7.13 (d, 1H, *J*= 7.1 Hz), 6.99 (d, 2H, *J*= 8.2 Hz, phenyl), 6.82 (t, 1H, *J*= 7.1 Hz, phenyl), 4.13 (m, 4H, piperazine), 3.32 (m, 4H, piperazine). ^{13}C-NMR (d_6-DMSO, 100 MHz, ppm):181.3, 153.8, 152.3, 151.0, 144.2, 138.6, 137.0, 129.5, 129.2, 128.6, 119.6, 118.3, 117.9, 115.9, 112.7, 50.4, 48.6. MP: 175-176°C. HRMS-ESI-TOF: 392.1540 $[M + H]^+$ ($C_{21}H_{22}N_5OS$; Exact Mass: 392.1545). Log P_{calc}: 3.833.

(*E*)-*N*'-((8-Hydroxyquinolin-2-yl)methylene)morpholine-4-carbothiohydrazide (3c)

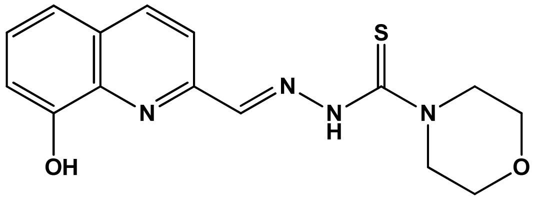

Yield: 76%. Purity: 95.77%. ^{1}H-NMR (d_6-DMSO, 400 MHz, ppm): 11.66 (bs, 1H, NH), 9.81 (bs, 1H, OH), 8.35 (s, 1H, CH), 8.30 (d, 1H, *J* = 8.7 Hz, quinoline), 7.97 (d, 1H, *J* = 8.7 Hz, quinoline), 7.45 (t, 1H, *J* = 7.7 Hz, quinoline), 7.39 (d, 1H, *J* = 7.6 Hz, quinoline), 7.12 (d, 1H, *J* = 7.1 Hz, quinoline), 3.98 (m, 4H, piperazine), 3.72 (m, 4H, piperazine). ^{13}C-NMR (d_6-DMSO, 100 MHz, ppm):181.4, 153.8, 152.2, 144.3, 138.6, 137.0, 129.1, 128.6, 118.3, 117.9, 112.6, 66.5, 51.2. MP: 181-182°C (181-182°C, [7]). HRMS-EI: 316.0984 ($C_{15}H_{16}N_4O_2S$; Exact Mass: 316.0994). Log P_{calc}: 1.841.

(*E*)-*N*'-((8-Hydroxyquinolin-2-yl)methylene)-4-(pyridin-2-yl)piperazine-1-carbothiohydrazide (3d)

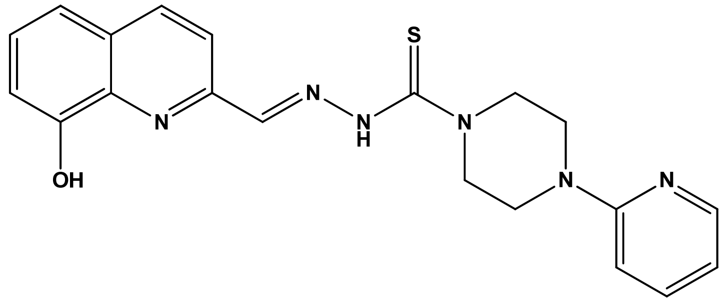

Yield: 77%. Purity: 99.25%. ^{1}H-NMR (d_6-DMSO, 400 MHz, ppm): 11.70 (bs, 1H, NH), 9.82 (bs, 1H, OH), 8.38 (s, 1H, CH), 8.31 (d, 1H, *J*= 8.7 Hz, quinoline), 8.16 (d, 1H, *J*= 4.8 Hz, pyridine), 8.02 (d, 1H, *J*= 8.7 Hz, quinoline), 7.58 (m, 1H, pyridine), 7.45 (t, 1H, *J*= 7.6 Hz, quinoline), 7.39 (d, 1H, *J*= 7.9 Hz, quinoline), 7.13 (d, 1H, *J*= 7.3 Hz, quinoline), 6.86 (d, 1H, *J*= 8.6 Hz, pyridine), 6.68 (m, 1H, pyridine), 4.11 (m, 4H, piperazine), 3.70 (m, 4H, piperazine). ^{13}C-NMR (d_6-DMSO, 100 MHz, ppm):181.3, 159.0, 153.8, 152.3, 148.0, 144.2, 138.6, 138.1, 137.0, 129.1, 128.6, 118.3, 118.0, 113.6, 112.6, 107.5, 50.2, 44.7. MP: 176-177°C. HRMS-ESI-TOF: 393.1490 $[M + H]^+$ ($C_{20}H_{21}N_6OS$; Exact Mass: 393.1498). Log P_{calc}: 2.885.

(*E*)-*N*'-((8-Hydroxyquinolin-2-yl)methylene)-4-(pyrazin-2-yl)piperazine-1-carbothiohydrazide (3e)

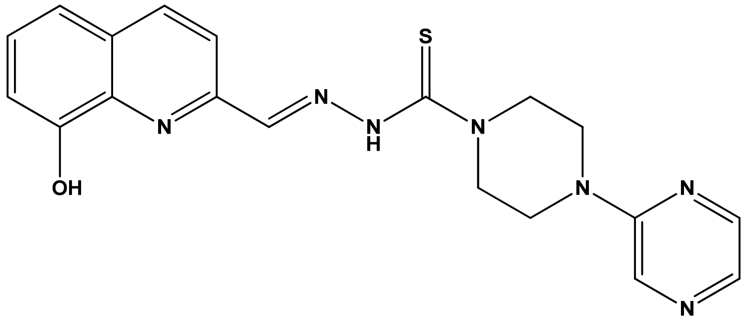

Yield: 86%. Purity: 99.8%. ^{1}H-NMR (d_6-DMSO, 400 MHz, ppm): 11.72 (bs, 1H, NH), 9.82 (bs, 1H, OH), 8.38 (s, 1H, CH), 8.36 (m, 1H, pyrazine), 8.31 (d, 1H, *J*= 8.7 Hz, quinoline), 8.13 (m, 1H, pyrazine), 8.02 (d, 1H, *J*= 8.7 Hz, quinoline), 7.89 (m, 1H, pyrazine), 7.45 (t,

1H, J = 7.6 Hz, quinoline), 7.39 (d, 1H, J= 7.5 Hz, quinoline), 7.13 (d, 1H, J= 6.7 Hz, quinoline), 4.13 (m, 4H, piperazine), 3.78 (m, 4H, piperazine). ^{13}C-NMR (d_6-DMSO, 100 MHz, ppm):181.4, 154.7, 153.8, 152.2, 144.3, 141.9, 138.6, 137.0, 133.1, 131.7, 129.2, 128.6, 118.3, 118.0, 112.6, 49.9, 44.0. MP: 200-201°C. HRMS-ESI-TOF: 394.1448 $[M + H]^+$ ($C_{19}H_{20}N_7OS$; Exact Mass: 394.1450). Log P_{calc}: 2.121.

(*E*)-*N*-Cyclohexyl-2-((8-hydroxyquinolin-2-yl)methylene)-N-methylhydrazinecarbothioamide (3f)

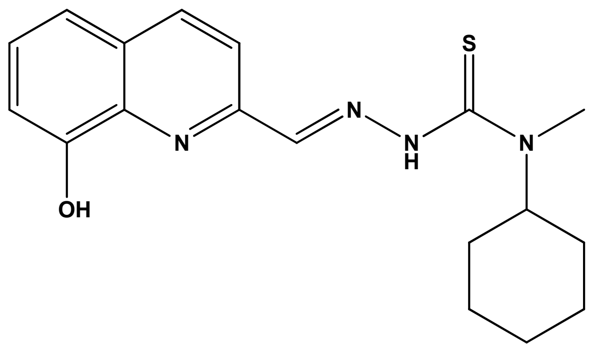

Yield: 65%. Purity: 98.01%. ^{1}H-NMR (d_6-DMSO, 400 MHz, ppm): 11.33 (bs, 1H, NH), 9.77 (bs, 1H, OH), 8.39 (s, 1H, CH), 8.30 (d, 1H, J= 8.8 Hz, quinoline), 7.99 (d, 1H, J= 8.8 Hz, quinoline), 7.44 (t, 1H, J= 7.6 Hz, quinoline), 7.38 (dd, 1H, J_1= 8.0 Hz; J_2= 1.2 Hz, quinoline), 7.11 (dd, 1H, J_1= 7.2 Hz; 1.2 Hz, quinoline), 4.74 (bs, 1H, C_1-cyclohexyl), 3.14 (s, 3H, CH_3), 1.80 (m, 4H, cyclohexyl),1.58 (m, 3H, cyclohexyl), 1.32 (m, 2H, cyclohexyl), 1.16 (t, 1H, J= 11.4 Hz, cyclohexyl). ^{13}C-NMR (d_6-DMSO, 100 MHz, ppm):181.2, 153.8, 152.5, 144.1, 138.6, 136.9, 129.1, 128.4, 118.3, 117.90, 112.6, 61.2, 39.8, 35.0, 29.7, 25.7, 25.4. MP: 164-165°C. HRMS-EI: 342.1513 ($C_{18}H_{22}N_4OS$; Exact Mass: 342.1514). Log P_{calc}: 4.193.

(*E*)-4-Ethyl-*N*'-((7-hydroxyquinolin-8-yl)methylene)piperazine-1-carbothiohydrazide (4a)

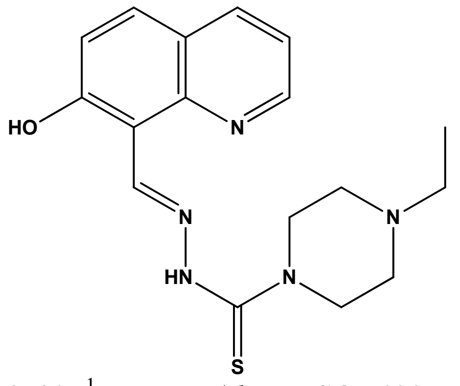

Yield: 76.5%. Purity: 95.35%. ^{1}H-NMR (d_6-DMSO, 400 MHz, ppm): 13.23 (bs, 1H, NH), 11.59 (bs, 1H, OH), 9.76 (s, 1H, CH), 8.84 (d, 1H; J = 2.8 Hz, quinoline), 8.30 (d, 1H; J = 8.1 Hz, quinoline), 7.92 (d, 1H, J = 9.0 Hz, quinoline), 7.42 (dd, 1H, J_1 = 8.1 Hz, J_2= 4.3 Hz, quinoline), 7.28 (d, 1H, J= 9.0 Hz, quinoline), 3.97 (m, 4H, piperazine), 2.51 (m, 6H, piperazine, CH_2), 1.05 (t, J= 7.2 Hz, 3H, CH_3). ^{13}C-NMR (d_6-DMSO, 100 MHz, ppm):179.2, 160.4, 150.6, 147.0, 144.9, 136.9, 131.6, 122.2, 120.8, 119.7, 111.0, 52.3, 51.7, 48.2, 12.1. MP: 145-146°C. HRMS-EI: 343.1464 ($C_{17}H_{21}N_5OS$; Exact Mass: 343.1467). Log P_{calc}: 2.931.

(*E*)-*N*'-((7-Hydroxyquinolin-8-yl)methylene)-4-phenylpiperazine-1-carbothio-hydrazide (4b)

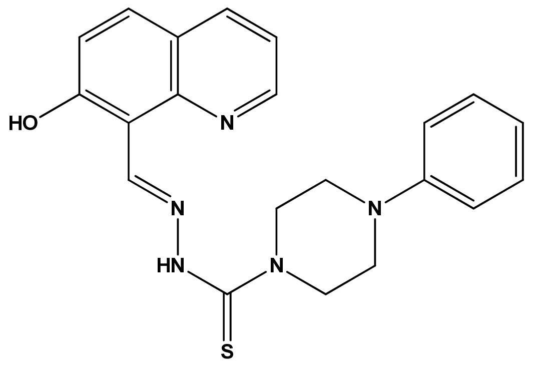

Yield: 67%. Purity: 96.12%. ^{1}H-NMR (d_6-DMSO, 400 MHz, ppm): 13.14 (bs, 1H, NH), 11.83 (bs, 1H, OH), 9.83 (s, 1H, CH), 8.87 (m, 1H, quinoline), 8.32 (d, 1H, J = 8.0 Hz, quinoline), 7.95 (d, 1H, J = 9.0 Hz, quinoline), 7.45 (dd, 1H, J_1 = 8.0 Hz, J_2= 4.2 Hz, quinoline), 7.31 (d, 1H, J = 8.9 Hz, quinoline), 7.25 (t, 2H, J = 7.7 Hz, phenyl), 7.00 (d, 2H, J

= 8.2 Hz, phenyl), 6.82 (t, 1H, *J* = 7.1 Hz, phenyl), 4.13 (m, 4H, piperazine), 3.29 (m, 4H, piperazine). ^{13}C-NMR (d_6-DMSO, 100 MHz, ppm):179.2, 159.8, 150.9, 150.7, 146.9, 145.2, 137.0, 131.6, 129.5, 122.4, 120.4, 119.9, 119.6, 115.9, 111.2, 48.3, 48.2. MP: 212-213°C. HRMS-EI: 391.1470 ($C_{21}H_{21}N_5OS$; Exact Mass: 391.1467). Log P_{calc}: 3.833.

(*E*)-*N'*-((7-Hydroxyquinolin-8-yl)methylene)morpholine-4-carbothiohydrazide (4c)

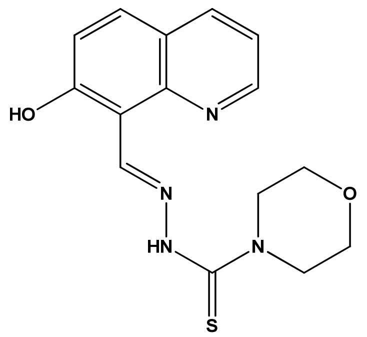

Yield: 76%. Purity: 96.22%. ^{1}H-NMR (d_6-DMSO, 400 MHz, ppm): 13.10 (bs, 1H, NH), 11.75 (bs, 1H, OH), 9.81 (s, 1H, CH), 8.87 (m, 1H, quinoline), 8.33 (d, 1H, *J* = 7.8 Hz, quinoline), 7.95 (d, 1H, *J* = 9.0 Hz, quinoline), 7.45 (dd, 1H, J_1 = 8.0 Hz, J_2= 4.3 Hz, quinoline), 7.31 (d, 1H, *J* = 8.9 Hz, quinoline), 3.97 (m, 4H, piperazine), 3.69 (m, 4H, piperazine). ^{13}C-NMR (d_6-DMSO, 100 MHz, ppm):179.5, 159.7, 150.7, 146.8, 145.3, 137.1, 131.6, 122.4, 120.4, 119.9, 111.2, 66.2, 49.1. MP: 198-199°C. HRMS-EI: 316.0982 ($C_{15}H_{16}N_4O_2S$; Exact Mass: 316.0994). Log P_{calc}: 1.841.

(*E*)-*N*'-((7-Hydroxyquinolin-8-yl)methylene)-4-(pyridin-2-yl)piperazine-1-carbothiohydrazide (4d)

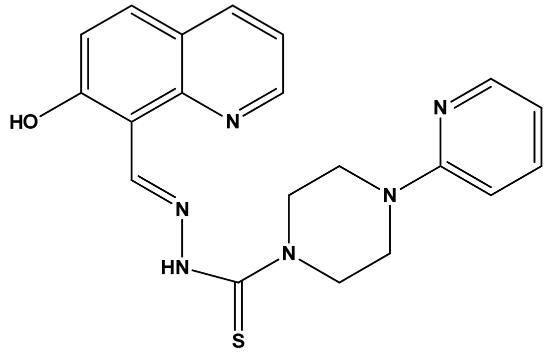

Yield: 72%. Purity: 95.29%. ^{1}H-NMR (d_6-DMSO, 400 MHz, ppm): 13.14 (bs, 1H, NH), 11.80 (s, 1H, OH), 9.83 (s, 1H, CH), 8.86 (dd, 1H, J_1 = 4.2, J_2= 1.6 Hz, quinoline), 8.32 (dd, 1H, J_1 = 8.2, J_2= 1.7 Hz, quinoline), 8.15 (dd, 1H, J_1 = 4.9, J_2= 1.6 Hz, pyridine), 7.95 (d, 1H, J = 9.0 Hz, quinoline), 7.58 (ddd, 1H, J_1 = 8.8, J_2=7.2, J_3= 1.9 Hz, pyridine), 7.45 (dd, 1H, J_1=8.2 Hz, J_2= 4.3 Hz, quinoline), 7.32 (d, 1H, J= 8.6 Hz, quinoline), 6.87 (d, 1H, J = 8.6 Hz, pyridine), 6.68 (dd, 1H, J_1 = 7.0, J_2= 5.0 Hz, pyridine), 4.11 (m, 4H, piperazine), 3.66 (m, 4H, piperazine). ^{13}C-NMR (d_6-DMSO, 100 MHz, ppm):179.2, 159.3, 158.9,150.6,148.0, 146.9, 145.2, 138.1, 137.0, 131.6, 122.3, 120.4, 119.8, 113.7, 111.9, 107.5, 56.5, 48.1. MP: 186-187°C. HRMS-ESI-TOF: 393.1493 $[M + H]^+$ ($C_{20}H_{21}N_6OS$; Exact Mass: 393.1498). Log P_{calc}: 2.885.

(*E*)-*N*'-((7-Hydroxyquinolin-8-yl)methylene)-4-(pyrazin-2-yl)piperazine-1-carbothiohydrazide (4e)

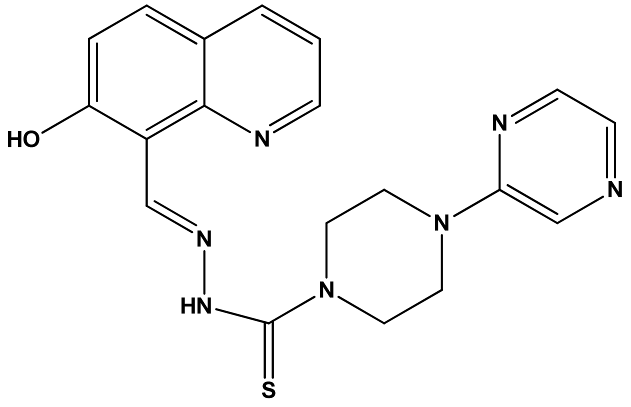

Yield: 76%. Purity: 98.37%. ^{1}H-NMR (d_6-DMSO, 400 MHz, ppm): 13.13 (bs, 1H, NH), 11.82 (bs, 1H, OH), 9.84 (s, 1H, CH), 8.87 (d, 1H, *J*= 2.5 Hz, quinoline), 8.37 (s, 1H, pyrazine), 8.33 (d, 1H, *J* = 7.9 Hz, quinoline), 8.13 (s, 1H, pyrazine), 7.95 (d, 1H, *J* = 9.0 Hz, quinoline), 7.89 (d, 1H, *J* = 2.3 Hz, pyrazine), 7.45 (dd, 1H, J_1 = 8.0, J_2= 4.2 Hz, quinoline), 7.32 (d, 1H, *J*= 8.4 Hz, quinoline), 4.13 (m, 4H, piperazine), 3.74 (m, 4H, piperazine). ^{13}C-NMR (d_6-DMSO, 100 MHz, ppm):179.3, 159.8, 154.7, 150.7, 146.9, 145.3, 141.9, 137.0, 133.1, 131.8, 131.6, 122.4, 120.4, 119.9, 111.2, 56.5, 47.8. MP: 209-210°C. HRMS-ESI-TOF: 394.1446 $[M + H]^+$ ($C_{19}H_{20}N_7OS$; Exact Mass: 394.1450). Log P_{calc}: 2.121.

(*E*)-*N*-Cyclohexyl-2-((7-hydroxyquinolin-8-yl)methylene)-*N*-methylhydrazine carbothioamide (4f)

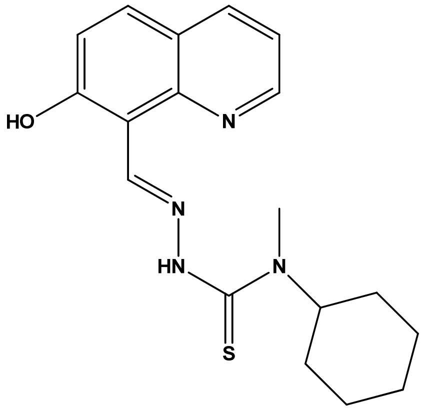

Yield: 76%. Purity: 95.46%. ^{1}H-NMR (d_6-DMSO, 400 MHz, ppm): 13.26 (bs, 1H, NH), 11.20 (bs, 1H, OH), 9.82 (s, 1H, CH), 8.85 (dd, 1H, J_1= 4.0 Hz; J_2= 1.6 Hz, quinoline), 8.30 (dd, J_1= 8.0 Hz, J_2= 1.9 Hz, quinoline), 7.92 (d, 1H, *J*= 9.0 Hz, quinoline), 7.43 (dd, 1H, J_1= 8.1 Hz, J_2= 4.3 Hz, quinoline), 7.29 (d, 1H, *J*= 9.0 Hz, quinoline), 5.00 (bs,1H, C_1-cyclohexyl), 3.10 (s, 3H, CH_3), 1.79 (m, 4H, cyclohexyl), 1.50 (m, 3H, cyclohexyl), 1.35 (m, 2H, cyclohexyl), 1.06 (t, 1H, *J*= 6.8 Hz, cyclohexyl). ^{13}C-NMR (d_6-DMSO, 100 MHz, ppm): 179.0, 160.1, 150.5, 147.0, 144.8, 136.9, 131.4, 122.3, 120.6, 119.7, 111.2, 59.5, 32.7, 29.6, 25.7, 25.4, 24.2. MP: 184-185°C. HRMS-EI: 342.1512 ($C_{18}H_{22}N_4OS$; Exact Mass: 342.1514). Log P_{calc}: 4.1935.

(*E*)-4-Ethyl-*N*'-(quinoxalin-2-ylmethylene)piperazine-1-carbothiohydrazide (5a)

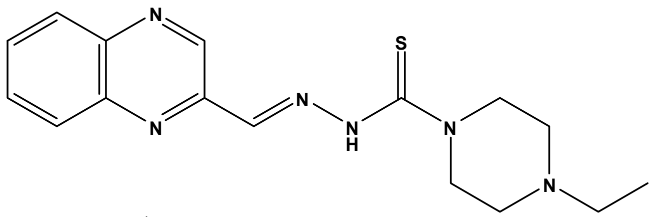

Yield: 86%. Purity: 96.00%. ^{1}H-NMR (d_6-DMSO, 500 MHz, ppm): 11.69 (bs, 1H, NH), 9.34 (s, 1H, quinoxaline), 8.32 (s, 1H, quinoxaline), 8.09 (t, 2H, *J* = 9.1 Hz, quinoxaline), 7.91 – 7.80 (m, 2H, quinoxaline), 3.98 (m, 4H, piperazine), 3.34 (m, 4H, piperazine), 2.39 (q, 2H, *J* = 7.1 Hz, CH_2), 1.04 (t, 3H, *J* = 7.2 Hz, CH_3). ^{13}C-NMR (d_6-DMSO, 125 MHz, ppm): 181.0, 149.1, 148.9, 143.2, 141.8, 141.8, 131.2, 130.9, 129.4, 129.4, 52.7, 51.8, 50.7, 12.4. MP: 150-151°C. HRMS-ESI-TOF: 329.1544 $[M + H]^+$ ($C_{16}H_{21}N_6S$; Exact Mass: 329.1548). Log P_{calc}: 2.136.

(*E*)-4-Phenyl-*N*'-(quinoxalin-2-ylmethylene)piperazine-1-carbothiohydrazide (5b)

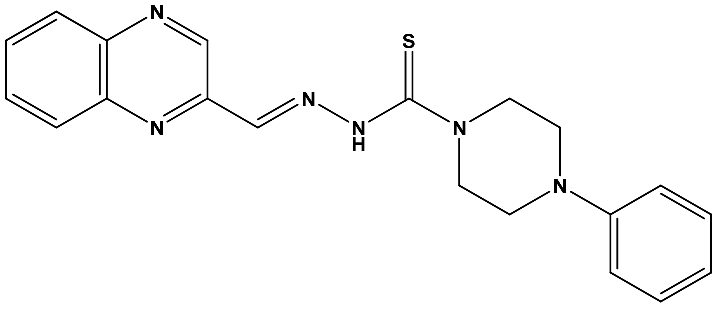

Yield: 69%. Purity: 99.10%. ^{1}H-NMR (d_6-DMSO, 400 MHz, ppm): 11.82 (bs, 1H, NH), 9.39 (s, 1H, quinoxaline), 8.36 (s, 1H; CH), 8.10 (t, 2H, *J*= 7.2 Hz, quinoxaline), 7.87 (m, 2H, quinoxaline), 7.26 (t, 2H, *J*= 8.0 Hz, phenyl), 7.00 (d, 2H, *J*=8.1 Hz, phenyl), 6.82 (t, 1H, *J*= 7.2 Hz, phenyl), 4.15 (m, 4H, piperazine), 3.33 (m, 4H, piperazine). ^{13}C-NMR (d_6-DMSO, 100 MHz, ppm): 181.1; 150.9; 149.1; 143.3; 142.0; 141.9; 141.8; 131.2; 130.9; 129.5; 129.5; 119.6; 115.9; 50.4; 48.5. MP: 168-169°C. HRMS-ESI-TOF: 377.1546 $[M + H]^+$ ($C_{20}H_{21}N_6S$; Exact Mass: 377.1548). Log P_{calc}: 3.038.

(*E*)-*N*'-(Quinoxalin-2-ylmethylene)morpholine-4-carbothiohydrazide (5c)

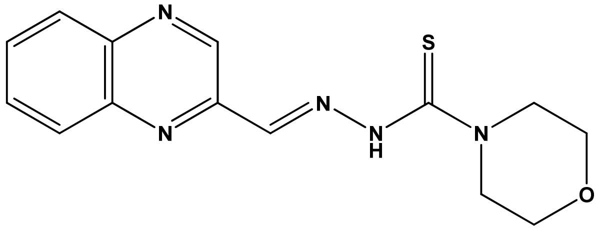

Yield: 83%. Purity: 99.00%. ^{1}H-NMR (d_6-DMSO, 400 MHz, ppm): 11.56 (bs, 1H, NH), 9.34 (s, 1H, quinoxaline), 8.33 (s, 1H, CH), 8.09 (m, 2H, quinoxaline), 7.85 (m, 2H, quinoxaline), 4.00 (m, 4H, piperazine), 3.73 (s, 4H, piperazine). ^{13}C-NMR (d_6-DMSO, 100 MHz, ppm): 181.3, 149.0, 143.3, 142.0, 141.8, 141.7, 131.2, 130.9, 129.4, 66.5, 51.3. MP: 176-177°C. HRMS-ESI-TOF: 324.0892 [M + Na]$^+$ ($C_{14}H_{15}N_5OSNa$; Exact Mass: 324.0895). Log P_{calc}: 1.046.

(*E*)-4-(Pyridin-2-yl)-*N*'-(quinoxalin-2-ylmethylene)piperazine-1-carbothiohydrazide (5d)

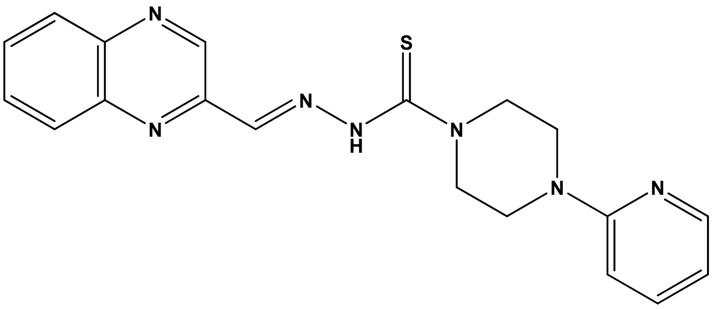

Yield: 69%. Purity: 98.45%. ^{1}H-NMR (d_6-DMSO, 400 MHz, ppm): 11.79 (s, 1H, NH), 9.39 (s, 1H, quinoxaline), 8.36 (s, 1H, CH), 8.15 (d, 1H, *J*= 3.2 Hz, pyridine), 8.10 (m, 2H, quinoxaline), 7.87 (m, 2H, quinoxaline), 7.58 (t, 1H, *J*= 7.2 Hz, pyridine), 6.85 (d, 1H, *J*= 8.0 Hz, pyridine), 6.68 (t, 1H, *J*= 5.6 Hz, pyridine), 4.12 (m, 4H, piperazine), 3.70 (m, 4H, piperazine). ^{13}C-NMR (d_6-DMSO, 100 MHz, ppm): 181.2, 159.0, 149.1, 148.1, 143.3, 141.9, 141.9, 141.8, 138.1, 131.2, 130.9, 129.5, 113.7, 107.5, 50.3, 44.7. MP: 190-191°C. HRMS-EI: 377.1405($C_{19}H_{19}N_7S$; Exact Mass: 377.1423). Log P_{calc}: 2.09.

(*E*)-4-(Pyrazin-2-yl)-*N'*-(quinoxalin-2-ylmethylene)piperazine-1-carbothiohydrazide (5e)

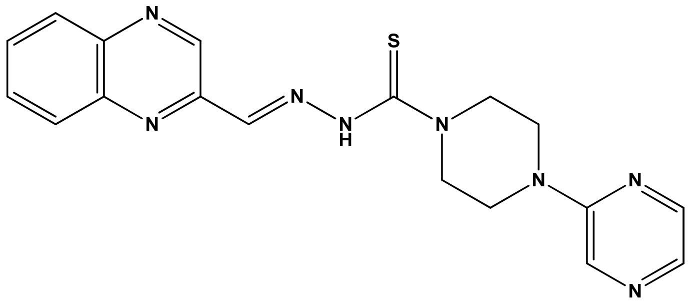

Yield: 79 %. Purity: 95.37%. ^{1}H-NMR (d_6-DMSO, 400 MHz, ppm): 11.81 (bs, 1H, NH), 9.40 (s, 1H, quinoxaline), 8.36 (m, 2H, CH, pyrazine), 8.13-8.08 (m, 3H, quinoxaline, pyrazine), 7.89-7.84 (m, 3H, quinoxaline, pyrazine), 4.15 (m, 4H, piperazine), 3.79 (m, 4H, piperazine). ^{13}C-NMR (d_6-DMSO, 100 MHz, ppm): 181.2, 154.7, 149.1, 143.3, 142.0, 141.9, 141.9, 141.8, 133.1, 131.7, 131.2, 130.9, 129.4, 129.3, 50.01, 44.0. MP: 186-187°C. HRMS-ESI-TOF: 401.1270 $[M + Na]^+$ ($C_{18}H_{18}N_8SNa$; Exact Mass: 401.1273). Log P_{calc}: 1.325.

(*E*)-*N*-Cyclohexyl-*N*-methyl-2-(quinoxalin-2-ylmethylene)hydrazinecarbothioamide (5f)

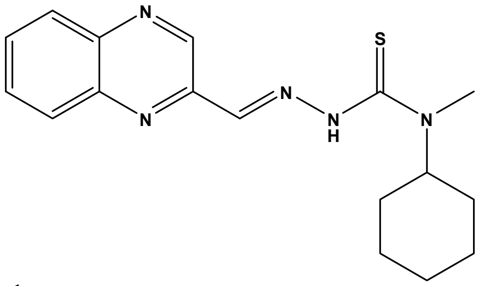

Yield: 78%. Purity: 98.70%. ^{1}H-NMR (d_6-DMSO, 400 MHz, ppm): 11.44 (bs, 1H, NH), 9.36 (s, 1H, quinoxaline), 8.38 (s, 1H, CH), 8.12-8.07 (m, 2H, quinoxaline), 7.88-7.85 (m, 2H, quinoxaline), 4.74 (bs, 1H, C_1-cyclohexyl), 3.16 (s, 3H, CH_3), 1.80 (m, 4H, cyclohexyl), 1.58 (m, 3H, cyclohexyl), 1.32 (m, 2H, cyclohexyl), 1.16 (t, 1H, J= 12.8 Hz, cyclohexyl). ^{13}C-NMR (d_6-DMSO, 100 MHz, ppm): 181.0, 149.3, 143.2, 141.9, 141.8, 131.2, 130.8, 129.4, 129.4, 61.3, 35.1, 29.7, 25.7, 25.4. MP: 154-155°C. HRMS-EI: 327.1513 ($C_{17}H_{21}N_5S$; Exact Mass: 327.1518). Log P_{calc}: 3.398.

(*E*)-4-Ethyl-*N*'-(2-hydroxybenzylidene)piperazine-1-carbothiohydrazide (6a)

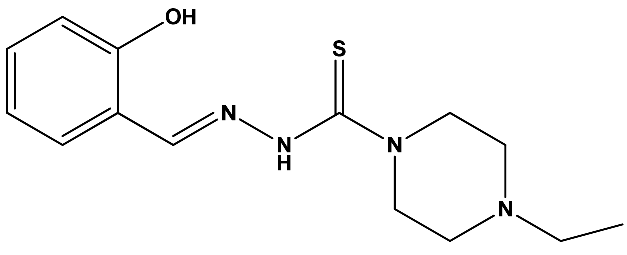

Yield: 86%. Purity: 95.2%. ^{1}H-NMR (d_6-DMSO, 400 MHz, ppm): 11.60 (bs, 1H, NH), 8.46 (s, 1H, CH), 7.41 (dd, 1H, J_1 = 7.9 Hz, J_2= 1.6 Hz, phenyl), 7.27 (td, 1H, J_1 = 7.8 Hz, J_2= 7.4 Hz, J_3= 1.7 Hz, phenyl), 6.92-6.88 (m, 2H, phenyl), 3.92 (m, 4H, piperazine), 2.45 (m, 4H, piperazine), 2.35 (m, 2H, CH_2), 1.03 (t, 3H, J = 7.2 Hz, CH_3). ^{13}C-NMR (d_6-DMSO, 100 MHz, ppm): 183.0, 159.0, 148.0, 138.0, 128.5, 121.2, 118.6, 117.5, 57.2, 44.4, 12.5. MP: 160-161°C (160-161°C, [7]). HRMS-ESI-TOF: 293.1434 $[M + H]^+$ ($C_{14}H_{21}N_4OS$; Exact Mass: 293.1436). Log P_{calc}: 2.832.

(*E*)-*N*'-(2-Hydroxybenzylidene)-4-phenylpiperazine-1-carbothiohydrazide (6b)

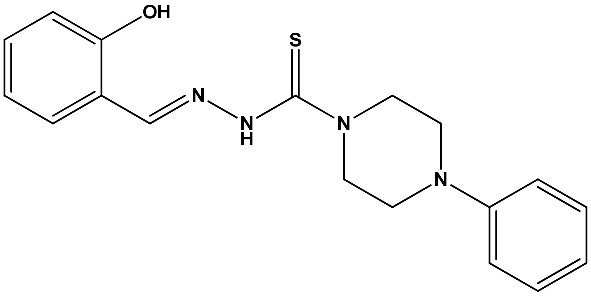

Yield: 89%. Purity: 96.12%. MP: 206-207°C [207-209°C; [4]]. Log P_{calc}: 3.231.

(*E*)-*N*'-(2-Hydroxybenzylidene)morpholine-4-carbothiohydrazide (6c)

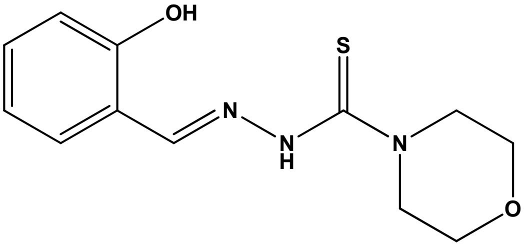

Yield: 91%. Purity: 95.96%. MP: 196-197°C [195°C; [4]]. Log P_{calc}: 1.239.

(*E*)-*N*'-(2-Hydroxybenzylidene)-4-(pyridin-2-yl)piperazine-1-carbothiohydrazide (6d)

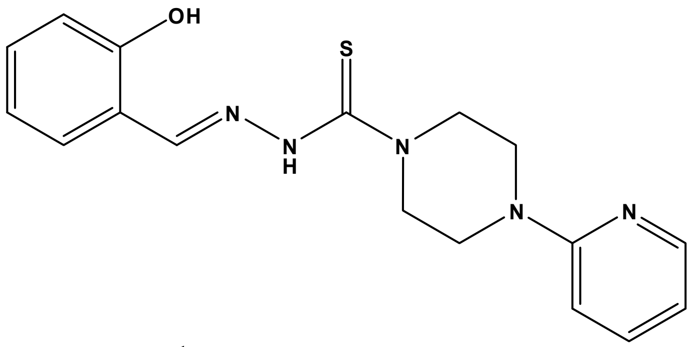

Yield: 88%. Purity: 99.3%. ^{1}H-NMR (d_6-DMSO, 400 MHz, ppm): 11.56 (bs, 1H, NH), 11. 54 (bs, 1H, OH), 8.50 (s, 1H, CH), 8.15 (dd, 1H, J_1 = 4.7 Hz, J_2= 1.4 Hz, pyridine), 7.58 (ddd, J_1 = 8.9 Hz, J_2= 7.2 Hz, J_3= 1.9 Hz, pyridine), 7.45–7.40 (m, 1H, phenyl), 7.32–7.24 (m, 2H, phenyl), 6.91 (dd, 1H, J_1= 7.7, J_2= 4.5 Hz, phenyl), 6.86 (d, 1H, J = 8.6 Hz, pyridine), 6.68 (dd, 1H, J_1 = 6.9 Hz, J_2= 5.0 Hz), 4.14–3.98 (m, 4H, piperazine), 3.67–3.59 (m, 4H, piperazine). ^{13}C-NMR (d_6-DMSO, 100 MHz, ppm): 179.8, 158.9, 157.6, 148.0, 146.8, 138.1, 131.3, 130.4, 119.5, 119.0, 117.0, 113.7, 107.5, 48.4, 44.4. MP: 191-192°C. HRMS-ESI-TOF: 342.1385 $[M+H]^+$ ($C_{17}H_{20}N_5OS$; Exact Mass: 342.1389). Log P_{calc}: 2.283.

(*E*)-*N*'-(2-Hydroxybenzylidene)-4-(pyrazin-2-yl)piperazine-1-carbothiohydrazide (6e)

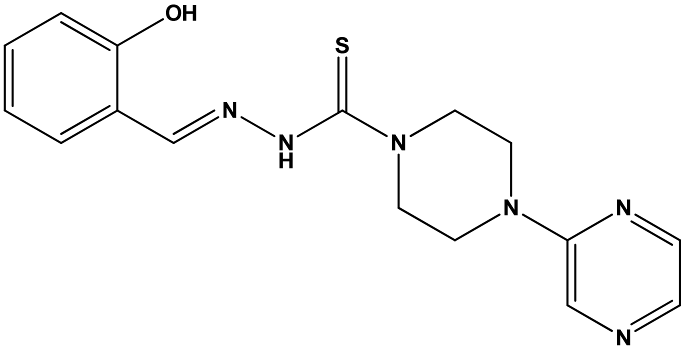

Yield: 89%. Purity: 98.9%. ^{1}H-NMR (d_6-DMSO, 400 MHz, ppm): 11.56 (bs, 1H, NH), 11. 54 (bs, 1H, OH), 8.51 (s, 1H, CH), 8.35 (d, 1H, J = 1.6 Hz, pyrazine), 8.12 (s, 1H, pyrazine), 7.88 (d, 1H, J = 2.6 Hz, pyrazine), 7.43 (dd, 1H, J_1 = 7.8 Hz, J_2= 1.7 Hz, phenyl), 7.28 (t, 1H, J= 8.0 Hz, phenyl), 6.97–6.82 (m, 2H, phenyl), 4.09 (m, 4H, piperazine), 3.73 (m, 4H, piperazine). ^{13}C-NMR (d_6-DMSO, 100 MHz, ppm): 179.8, 157.6, 154.7, 141.9, 133.1, 131.8,

131.4, 130.4, 119.6, 119.0, 117.0, 48.1, 43.7. MP: 189-190°C. HRMS-ESI-TOF: 343.1343 ($C_{16}H_{19}N_6OS$; Exact Mass: 343.1341). Log P_{calc}: 1.518.

(*E*)-*N*-Cyclohexyl-2-(2-hydroxybenzylidene)-*N*-methylhydrazinecarbothioamide (6f)

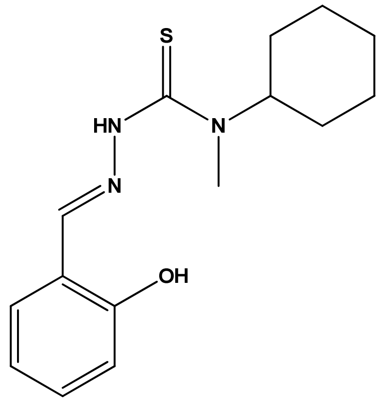

Yield: 74%. Purity: 95.89%. ^{1}H-NMR (*d$_6$*-DMSO, 400 MHz, ppm): 11.73 (bs, 1H, NH), 11.13 (bs, 1H, OH), 8.52 (s, 1H, CH), 7.38 (dd, 1H, J_1 = 7.8, J_2= 1.4 Hz, 1H, phenyl), 7.27 (t, 1H, *J*= 7.6 Hz, phenyl), 6.94–6.85 (m, 2H, phenyl), 4.98 (bs, 1H, C_1-cyclohexyl), 3.07 (s, 3H, CH_3), 1.79 (m, 4H, cyclohexyl), 1.50 (m, 3H, cyclohexyl), 1.29 (m, 2H, cyclohexyl), 1.14 (t, 1H, *J* = 15.4 Hz, cyclohexyl). ^{13}C-NMR (*d$_6$*-DMSO, 100 MHz, ppm): 179.4, 157.6, 146.8, 131.2, 130.6, 119.5, 118.9, 117.0, 59.8, 32.9, 29.5, 25.7, 25.4. MP: 145-146°C. HRMS-EI: 291.1401 ($C_{15}H_{21}N_3OS$; Exact Mass: 291.1405). Log P_{calc}: 3.591.

S1.2 X-ray data for selected thiosemicarbazones and thiosemicarbazides

X-ray crystal data were collected on an Oxford Diffraction Gemini A Ultra diffractometer using graphite monochromated Mo Kα radiation at a temperature of 295.0(2) K with x scan mode. Polarization, lorentz and empirical absorption corrections were applied using spherical harmonics implemented in the SCALE3 ABSPACK scaling algorithm (CrysAlis RED, Oxford Diffraction Ltd., Version 1.171.29.2). The structures were solved by direct methods and subsequently completed from Fourier difference recycling. All the non-hydrogen atoms were refined anisotropically using full-matrix, least-squares analysis. All hydrogen atoms were located from the difference maps after four cycles of anisotropic refinement, and refined using a riding model. The OLEX2 [8], SHELXS97 and SHELXL97 [9] programs were used for all calculations. All data were deposited with the Cambridge Crystallographic Data Center (CCDC 918418 & 919683).

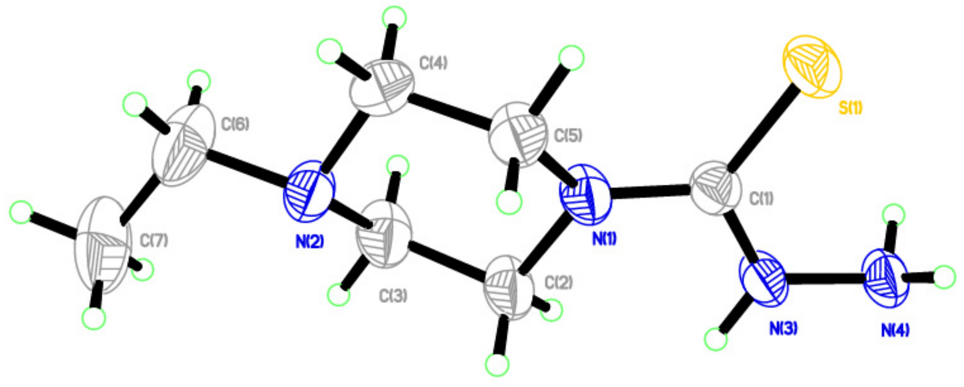

Figure S1. The crystal structure of 4-ethylpiperazine-1-carbothiohydrazide (**a**).

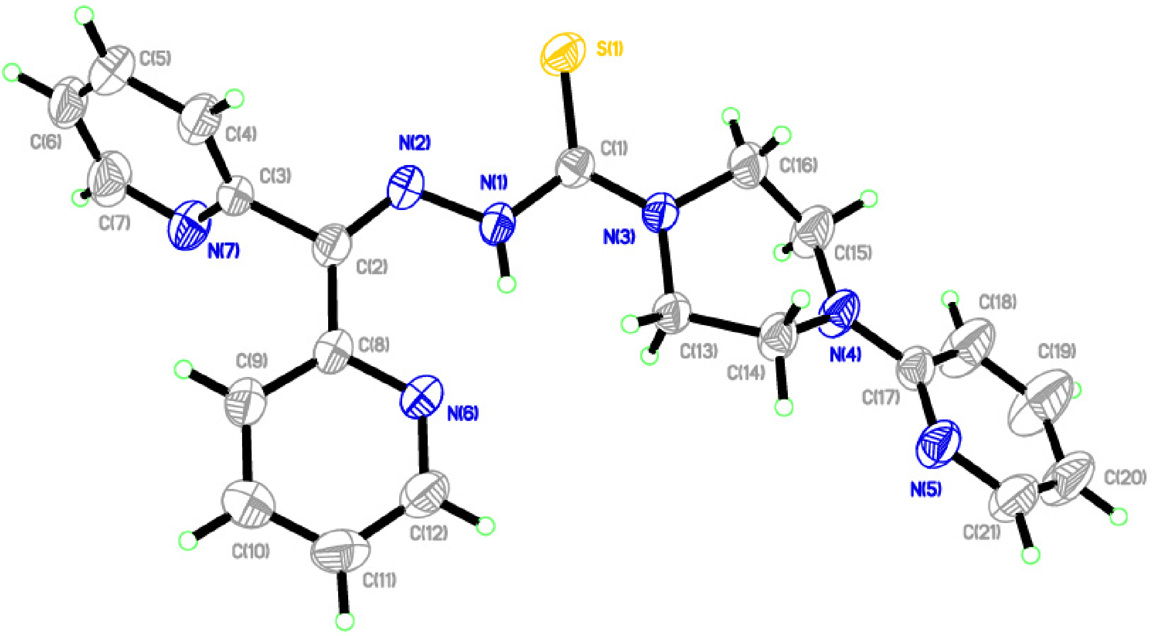

Figure S2. The crystal structure of *Z*-*N'*-(di(pyridin-2-yl)methylene)-4-(pyridin-2-yl)piperazine-1-carbothiohydrazide (**1d**).

Table S1. Crystal data of **a** and **1d**.

	4-Ethylpiperazine-1-carbothiohydrazide (**a**)	*Z*-*N'*-(Di(pyridin-2-yl)methylene)-4-(pyridin-2-yl)piperazine-1-carbothiohydrazide (**1d**)
**Formula**	$C_7H_{16}N_4S$	$C_{21}H_{21}N_7S$
**Formula weight**	188.3	403.51
**Crystal system**	Monoclinic	Triclinic
**Space group**	*C*2/*c*	$P\bar{1}$
**Color**	White	Yellow
**a (Å)**	12.3069(7)	9.2850(6)
**b (Å)**	8.0115(4)	9.5201(8)
**c (Å)**	21.0221(12)	11.6512(7)
**α (°)**	90	76.265(6)
**β (°)**	101.825(6)	88.780(5)
**γ (°)**	90	79.734(6)
**V (Å^3)**	2028.72(19)	984.19(12)
**T (K)**	295	295
**D_{Calc} (mg/m^3)**	1.233	1.362
**Z**	8	2
**R_1 (obsd data)**	0.0350(1490)	0.0444(2603)
**wR_2 (all data)**	0.0934(1789)	0.1174(3480)
**CCDC no.**	918418	919683

S1.3 HPLC purity data

Table S2. HPLC purity data for all chelators of series **1-6**.

Compound	t_R (min)	Purity (%)
**1a**	2.34	97.32
**1b**	2.07	98.65
**1c**	2.15	96.32
**1d**	2.15	95.62
**1e**	2.25	95.45
**2a**	1.98	96.67
**2b**	1.96	98.00
**2c**	2.0	97.42
**2d**	2.05	97.87
**2e**	2.02	98.98
**2f**	1.98	99.12
**3a**	2.4	95.03
**3b**	2.5	96.59
**3c**	2.52	95.77
**3d**	2.39	99.25
**3e**	2.53	99.8
**3f**	2.56	98.01
**4a**	2.0	95.35
**4b**	2.01	96.12
**4c**	2.07	96.22
**4d**	1.98	98.00
**4e**	2.1	98.37
**4f**	2.01	95.46
**5a**	1.99	96.00
**5b**	1.98	99.10
**5c**	2.0	99.00
**5d**	2.05	98.45
**5e**	2.11	95.37
**5f**	1.98	98.70
**6a**	2.54	95.2
**6b**	1.96	96.12
**6c**	2.00	95.96
**6d**	2.02	99.3
**6e**	2.06	98.9
**6f**	1.97	95.89

S1.4 Isosbestic curves

The ligand and complexes were prepared by dissolving the ligand (0.1 mM) in DMSO and various concentrations of $FeCl_3$ were added to obtain the following ligand:Fe^{3+} ratios: 1:1, 2:1, 4:1, 5:1, and 10:1. The collected spectra were overlaid to observe isosbestic points.

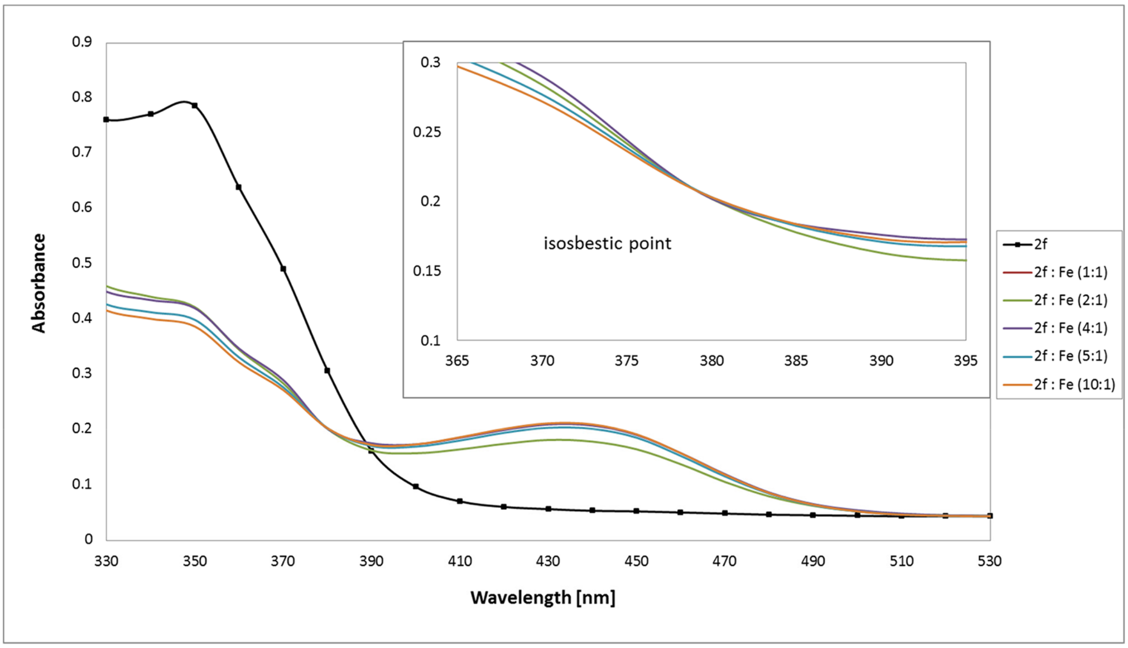

Figure S3. The absorbance spectrum of **2f** and its Fe^{3+} complexes prepared *in situ* to obtain 1:1, 2:1, 4:1, 5:1, and 10:1 ligand:Fe ratios.

S2 Anti-proliferative activity color maps

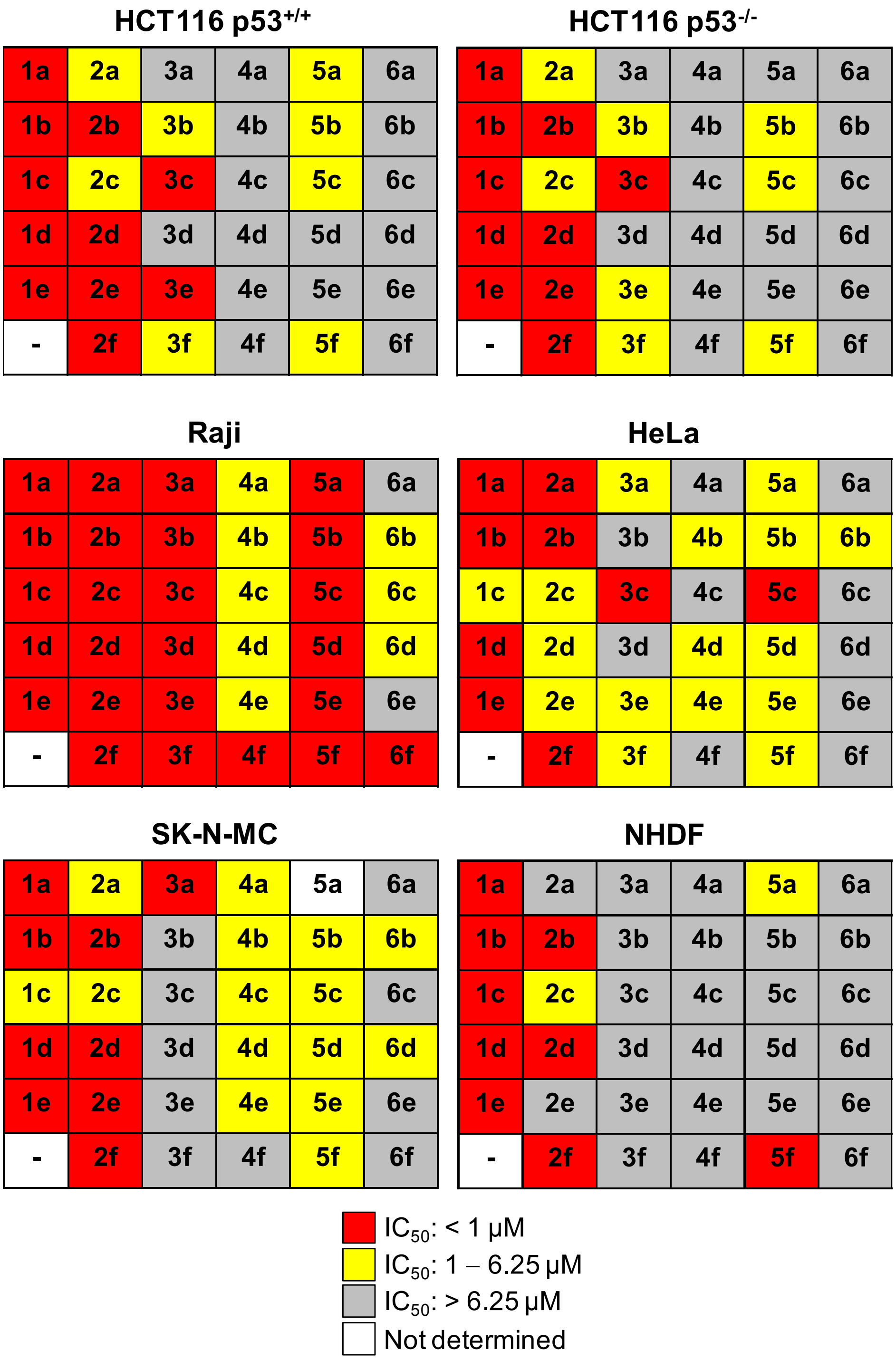

Figure S4. Color maps of the anti-proliferative activity of series **1-6** in several tumor cell-types and normal human dermal fibroblast (NHDF) cells. Red represents the thiosemicarbazones with the greatest anti-proliferative activity (IC_{50}: <1 μM), yellow represents the thiosemicarbazones with moderate activity (IC_{50}: 1 – 6.25 μM) and grey represents those analogs with poor anti-proliferative effects (IC_{50} > 6.25 μM).

S3 References

1. Ghose AK, Crippen GM (1987) Atomic physicochemical parameters for three-dimensional-structure-directed quantitative structure−activity relationships. 2. Modeling dispersive and hydrophobic interactions. J Chem Inf Comput Sci 27: 21-35.
2. Viswanadhan VN, Ghose AK, Revankar GR, Robins RK (1987) Atomic physiochemical parameters for 3-dimensional-structure directed quantitative structure−activity relationships 4. Additional parameters for hydrophobic and dispersive interactions and their application for an automated superposition of certain naturally-occuring nucleoside antibiotics. J Chem Inf Comput Sci 29: 163−172.
3. Broto P, Moreau G, Vandycke C (1984) Molecular structures: Perception, autocorrelation descriptor and SAR studies. System of atomic contributions for the calculation of the n-octanol/water partition coefficients. Eur J Med Chem Chim Theor 19: 71-78.
4. Spalinska K, Foks H, Kedzia A, Wierzbowska M, Kwapisz E, et al. (2006) Synthesis and antibacterial activity of substituted thiosemicarbazides and of 1,3,4-thiadiazole or 1,2,4-triazole derivatives. Phospho Sulfur 181: 609-625.
5. Milczarska B, Foks H, Zwolska Z (2005) Studies on pyrazine derivatives, XLII: Synthesis and tuberculostatic activity of 6-(1,4-dioxa-8-azaspiro-[4, 5]-decano)- and 6-(1-ethoxycarbonylpiperazino)-pyrazinocarboxylic acid derivatives. Phospho Sulfur 180: 1977-1992.
6. Lovejoy DB, Sharp DM, Seebacher N, Obeidy P, Prichard T, et al. (2012) Novel second-generation di-2-pyridylketone thiosemicarbazones show synergism with standard chemotherapeutics and demonstrate potent activity against lung cancer xenografts after oral and intravenous administration in vivo. J Med Chem 55: 7230-7244.
7. Mrozek-Wilczkiewicz A, Serda M, Musiol R, Malecki G, Szurko A, et al. (2014) Iron chelators in photodynamic therapy revisited: Synergistic effect by novel highly active thiosemicarbazones. ACS Med Chem Lett 5: 336-339.

8. Dolomanov OV, Bourhis LJ, Gildea RJ, Howard JAK, Puschmann H (2009) OLEX2: A complete structure solution, refinement and analysis program. J Appl Cryst 42: 339-341.

9. Sheldrick GM (2008) A short history of SHELX. Acta Cryst A64: 112-122.
